# Female Fertilization: Effects of Sex-Specific Density and Sex Ratio Determined Experimentally for Colorado Potato Beetles and *Drosophila* Fruit Flies

**DOI:** 10.1371/journal.pone.0060381

**Published:** 2013-04-12

**Authors:** Wouter K. Vahl, Gilles Boiteau, Maaike E. de Heij, Pamela D. MacKinley, Hanna Kokko

**Affiliations:** 1 Laboratory of Ecological & Evolutionary Dynamics, Department of Biosciences, Helsinki University, Helsinki, Finland; 2 Evolution, Ecology, & Genetics, Research School of Biology, Australian National University, Canberra, Australia; 3 Agriculture & Agri-food Canada, Fredericton, New Brunswick, Canada; 4 Bird Ecology Unit, Department of Biosciences, Helsinki University, Helsinki, Finland; CNRS, Université de Bourgogne, France

## Abstract

If males and females affect reproduction differentially, understanding and predicting sexual reproduction requires specification of response surfaces, that is, two-dimensional functions that relate reproduction to the (numeric) densities of both sexes. Aiming at rigorous measurement of female per capita fertilization response surfaces, we conducted a multifactorial experiment and reanalyzed an extensive data set. In our experiment, we varied the density of male and female *Leptinotarsa decemlineata* (Colorado potato beetles) by placing different numbers of the two sexes on enclosed *Solanum tuberosum* (potato plants) to determine the proportion of females fertilized after 3 or 22 hours. In the reanalysis, we investigated how the short-term fertilization probability of three *Drosophila* strains (*melanogaster ebony*, *m. sepia*, and *simulans*) depended on adult sex ratio (proportion of males) and total density. The fertilization probability of female *Leptinotarsa decemlineata* increased logistically with male density, but not with female density. These effects were robust to trial duration. The fertilization probability of female *Drosophila* increased logistically with both sex ratio and total density. Treatment effects interacted in *m. sepia*, and *simulans*. These findings highlight the importance of well-designed, multifactorial experiments and strengthen previous experimental evidence for the relevance of sex-specific densities to understanding and prediction of female fertilization probability.

## Introduction

How does reproductive success relate to sex-specific densities, that is, to the number of males and females in a certain area? Simple as the question may be, its answer is not obvious. Successful reproduction depends on a sequence of events (e.g. mate encounter, mate choice, mating) resulting from complex social behaviour (e.g. male-male competition, female harassment by males, parental care) that may be affected differentially by the densities of the two sexes [1]. The question is pertinent for at the core of studies of sexual reproduction, its genetics, causes, consequences, or evolution, lie (implicit or explicit) assumptions about density-dependence [2]. If male density and female density affect reproductive success differentially, knowing the total density of individuals is not sufficient to understand and predict their reproductive success. In that case, knowledge of the sex-specific densities or of the frequency of the two sexes (the tertiary, adult or operational sex ratio) is required too.

Numerous empirical studies support the notion that male and female density can affect reproductive success differentially. Drawing quantitative inferences from the available evidence is, however, not straightforward, because evidence is scattered. Relevant experiments have addressed the subject in the contexts of mating kinetics (e.g. [3]–[5]), sexual conflict (e.g. [6], [7]), sexual selection (e.g. [8], [9]), animal breeding (e.g. [10], [11]), population dynamics (e.g. [12], [13]), and pest control (e.g. [14], [15]). Methodologically, these experiments differ on important aspects, including the amount of (experimental and statistical) control exerted, the temporal and spatial scale of measurement, subtleties of design and analysis, and the response measure used to quantify reproductive success.

Undoubtedly, much is to be gained from a thorough, quantitative review that brings together and scrutinizes the available evidence in the light of methodological differences. Ultimately, however, quantitative inference from the evidence currently available will be hampered in at least three significant ways. First, treatment effects have generally not been determined at a wide range of multiple levels of independently varied, orthogonal treatment factors (but see for instance [16]–[18]). This makes parameter estimates potentially biased or imprecise and may limit the generality of conclusions [19], [20]. Second, few studies have determined the joint (i.e. interaction) effects of the density of males and females (but see for instance [6], [21], [22]). This makes conclusions potentially incomplete [23]. Third, few of the studies determined the relationship at issue quantitatively (but see for instance [7], [24], [25]). This requires (multiple) regression analysis, in which treatment factors are treated as continuous [20], [23]; in most studies treatment factors were instead treated as categorical.

To overcome these inferential problems, it seems worthwhile to reanalyze some of the previous experimental work, but also to collect new evidence from experiments designed and analyzed specifically to quantify ‘response surfaces’ [20], [23], that is, the two-dimensional functions that relate measures of reproductive success to the density of males and females. The latter approach was recently taken by Miller and Inouye [26], who quantified response surfaces of recruitment to total density and sex ratio.

Here, our approach is two-fold: we present a novel experiment and reanalyse an experimental data set available in the literature [3], [18]. Throughout this document, we measure reproductive success as female probability of fertilization. Fertilization is obviously a prerequisite for (sexual) reproduction and its probability can be measured almost instantaneously. Several experimental studies, together covering a wide range of species, have examined how, qualitatively, sex-specific densities affect female fertilization probability (for an overview, see Appendix S1). Almost without exception, these experiments found either no effects, or, at least for part of the range examined, positive effects of male density and sex ratio (proportion of males) as well as negative effects of female density.

In our experiment, we used a multifactorial design to systematically investigate how, in the course of a few hours, the fertilization probability of female *Leptinotarsa decemlineata* (Colorado potato beetles) depends on the on-plant density of males and females. We chose our study species because of its well-documented natural history (e.g. [27], [28]), economic significance (e.g. [29]), and the proven feasibility of density-dependence experiments (e.g. [30], [31]). The small spatial scale of our experiment, and the short temporal scale seemed relevant for this species given frequent and short-lasting matings (e.g. [32]) and large variation in density and sex ratio on individual field plants (e.g. Appendix S2).

For our reanalysis, we chose the data of the mating kinetics of *Drosophila* (fruit flies) in Wallace [3], [18] because of the unprecedented scale of these measurements. Wallace related the proportion of females fertilized to 21 experimentally-determined combinations of male density and female density in each of 13 experiments with together over 3390 trials. Furthermore, Wallace had the foresight to provide the full data set “for the benefit of those readers who wish to ask other questions of the data or to test hypotheses of their own making” [18]. In our reanalysis, we investigated: (1) how the fertilization probability of female *Drosophila melanogaster sepia* depended on the sex ratio and the total density of flies in a single mating chamber (data from Wallace [3]), and (2) whether treatment effects were robust to variation in duration of trials, number of mating chambers, and strain (data from Wallace [18]). Reanalysis in terms of fertilization probability is justified because Wallace mainly focused on attrition rate and male-male interactions.

## Methods

We first present the methods of our experiment with *Leptinotarsa decemlineata*, after which we summarize the *Drosophila* experiments of Wallace [3], [18] and present our reanalysis.

### Leptinotarsa decemlineata experiment

The experiment, conducted in a climatically controlled environment, consisted of two runs of 36 trials each. In each trial, we brought together a given number of male and female beetles (six levels each: 1, 2, 4, 8, 16, 32) on enclosed potato plants (*Solanum tuberosum*). After a few hours we collected all beetles to determine the proportion of females fertilized (i.e. inseminated); we varied the duration of trials between the two runs to examine the robustness of treatment effects. Keeping the duration of trials short and enclosing study subjects helped to approximate the direct effects at issue by minimizing density changes due to mortality, dispersal, and diapausing.

#### Subjects

We used summer-adult-population beetles, collected in July 2008 as 4^th^ instar larvae from an untreated potato field of the AAFC Potato Research Center in Fredericton, New Brunswick, Canada. We raised these larvae such that we could ensure their virginity as adults (see Appendix S3). The 756 male and the 756 female beetles used in the experiment were all between 8 and 13 days (inclusive) old, ensuring sexual maturity [33].

#### Set-up and procedure

We conducted six trials of the same duration on each of 12 days (4 to 15 August 2008). All trials were preceded by a 1 h acclimation period, in which same sex beetles could distribute themselves over two potato plants that we had placed in pre-selected cages (for more detail, see Appendix S3). We initiated each trial by carefully transferring plants with male beetles to an experimental cage (measuring 57×57 cm and 60 cm high) containing plants with female beetles. At the end of each trial we collected the beetles, noting down how many beetles were in tandem formation, on the cage, or on its floor. To prevent further mating, we killed all beetles instantaneously in 90% ethanol, and then stored them dry in a freezer (at −18°C±2°C) to await dissection. Upon dissection, we checked the sex of all beetles, and we examined the contents of spermatheca in females to detect fertilization. Presence of sperm was determined visually from water-based preparations, using a Zeiss IM35 inverted microscope (enlargement: 63 to 1000×); a cloud of milky white, sometimes light pink substance coming out of a spermathecum upon light pressure was taken to be sperm, especially when (in cases of doubt) zooming in revealed this substance to consists of very many needle-like elements.

#### Design

**Figure 1:**
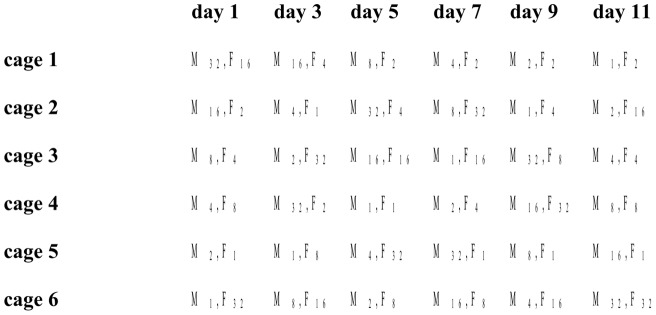
Schematic depiction of the design of the experimental run in which trial duration was ‘long’. Depicted is how, approximating a Graeco-Latin Square, each of the 36 unique combinations of the six levels of male density ‘M’ and female density ‘F’ were laid out over the six levels of the block factors experimental day and cage. The 36 trials of the experimental run in which trial duration was ‘short’ were laid out over the same cages but six other experimental days (days 2, 4, 6, 8, 10 and 12) in a similar manner, although their exact distribution was different.

In both runs of the experiment, we used a multifactorial design to determine how female fertilization probability was affected by the factors male density and female density, each with six treatment levels (for justification of these treatment levels, see Appendix S3). To account for variation between days and cages, trials were laid out according to a design that approximated a Graeco-Latin Square ([23]; see Fig. 1 for a schematic depiction). Male density was fully balanced with regard to female density, day and cage, such that the six male density levels featured once on each of six days, in each of six cages, and together with each of the six female density levels. Female density was similarly balanced with regard to day and male density, but not with regard to cages; some female density levels did not feature two times in each cage (range: 0 to 5). As a consequence, full separation of effects of female density and cage was not possible.

To facilitate comparison, we conducted the two runs of the experiment on alternating days. The two runs differed in the duration of trials, trial duration being either ‘short’ (3 h) or ‘long’ (22 h)(for justification of these levels, see Appendix S3).

#### Statistical analysis

We used logistic regression to analyze treatment and interaction effects on logit-transformed ‘fertilization probability’ (measured as the proportion of females fertilized). We treated ‘male density’ and ‘female density’ as continuous and fixed factors. To improve the spread of treatment levels we log_2_-transformed their values. We treated the block factors ‘day’ and ‘cage’ both as categorical random factors, normally distributed around zero. To increase statistical power we analyzed the two runs of the experiment together, including ‘trial duration’ as an additional continuous, fixed factor that varied between plots (days). We nested days within trial duration by labelling all days uniquely. The analyses included all possible interaction terms between fixed factors. To avoid correlations between component (main) effects and interaction effects, we centered all factors [34]. The strongest correlation for any combination of centered, fixed model terms was 0.01 (r_Pearson_, absolute value). There were few missing values; for a description of how we dealt with them and for more detail on the statistical analysis, see Appendix S3.

To evaluate treatment effects on female fertilization probability, we implemented our model in R [35] using the ‘lmer’ function for generalized linear mixed effects models [36]. Because the experiment contained only few factors, and because these factors were orthogonal, we focused our analysis on the full model only (conform [37], §4.12). We evaluated the performance of this model thoroughly (guided mainly by chapter 5 of Collett [38]), and we examined the extent to which parameter estimates depended upon model assumptions extensively through alternative analyses (see Appendix S3).

Note that our model does not consider effects of total density and sex ratio. We did not include these compound variables, because they were (inevitably) highly correlated with male density and female density (maximum r_Pearson_ 0.71). Instead, we evaluated effects of ‘total density’ (the sum of male density and female density) and ‘sex ratio’ (the proportion of males) in a separate model; composition and results of this model are presented in Appendix S4.

Aware of the limitations of and pitfalls associated with hypothesis testing (e.g. [39]), we summarize test results by presenting estimates of model parameters, their standard error and confidence intervals, as well as other quantities of interest (z-values and odd ratios), but not p-values.

### Drosophila experiment of Wallace

**Figure 2:**
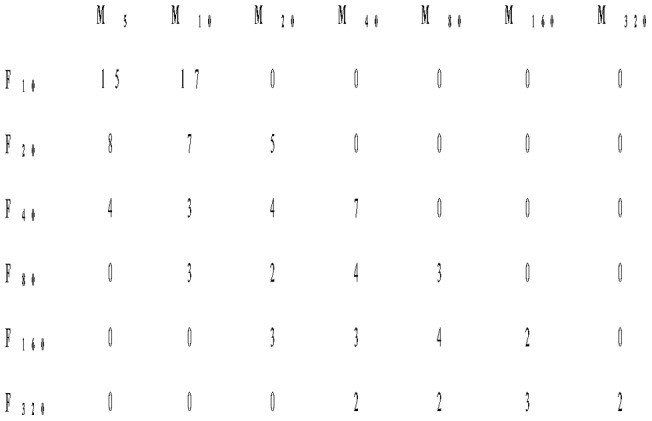
Schematic depiction of the experimental design used in Wallace [**3**]. Depicted is the minimum number of trials performed for each of the combinations of six levels of the number of females ‘F’ and seven levels of the number of males ‘M’.

Wallace [3] presents one experiment of at least 103 trials in which given numbers of male and female *D. m. sepia* were brought together in single mating chambers (∼2.8 dm^3^). His trials lasted for 30 minutes, after which females were isolated to determine the proportion of females fertilized (producing offspring). An incomplete multifactorial design (for a schematic depiction, see Fig. 2) was laid out to determine the effect of the number of males (7 levels: 5, 10, 20, 40, 80, 160, 320 individuals) and females (6 levels: 10, 20, 40, 80, 160, 320 individuals) on offspring production. Due to restrictions imposed on the sex ratios used in the experiments (female-biased sex ratios and extremely male-biased sex ratios were not examined), the design was unbalanced, with considerable correlation between male density and female density.

**Figure 3:**
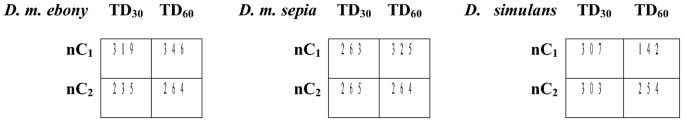
Schematic depiction of the experimental design used in Wallace [**18**]. Depicted is, for each of the three *Drosophila* strains, the minimum number of trials performed per combination of the whole plot factors number of cages ‘nC’ and trial duration ‘TD’. For each combination of these two factors (each cell), the same combinations of the subplot factors number of males and number of females as in Wallace ([3]; see Fig. 2) were examined.

Wallace [18] presents twelve similar experiments of together at least 3287 trials (minimally 142 to 346 trials per experiment). Experiments in Wallace [18] differ from those in Wallace [3] with respect to the study strain (*D. m. ebony*, *D. m. sepia*, *D. simulans*), the number of mating chambers (1 or 2), and the duration of trials (30 or 60 minutes). These three additional treatments were laid out across experiments to yield a five-factorial split-plot design (for a schematic depiction, see Fig. 3) that was fully balanced for study strain, trial duration and number of mating chambers at the whole plot level, but unbalanced for male density and female density at the subplot level.

#### Statistical reanalysis

Reanalyzing the data of Wallace [3], we followed the analysis of our own experiment as closely as possible. However, because in this study ‘sex ratio’ (proportion of males: 4 levels) and ‘total density’ (males plus females: 21 levels) were correlated to a considerably lesser extent (r_Pearson_  = 0.10 and -0.02 before and after log_2_-transformation, of total density, respectively) than male density and female density (r_Pearson_  = 0.69 and 0.79 before and after log_2_-transformation, respectively), we now ran a model focusing on the former two treatment factors. We treated sex ratio, total density, and their interaction as continuous and fixed factors. We log_2_-transformed the treatment levels of total density, left the treatment levels of sex ratio untransformed, and centered both factors. The strongest correlation between any two centered model terms was 0.25 (absolute value).

For data contained in Wallace [18], we first conducted exactly the same analysis as described above for data from Wallace [3] for each of the twelve experiments separately. To study whether treatments effects were robust with regard to variation in the number of mating chambers, trial duration, and study strain, we then extended the analysis with the latter three variables. In doing so, we treated the ‘number of mating chambers’ and ‘trial duration’ as continuous and fixed factors, and ‘study strain’ as a categorical and fixed factor. We did not transform the extra two continuous factors, but centered them to minimize correlations (the maximum correlation between any two terms in the resulting model was still 0.25). Because the number of mating chambers varied between experiments, total density was now varied in two independent ways: through the ‘total *number* of flies’ (note the change in terminology) and through the number of mating chambers. All possible interactions between the five fixed treatment factors were included in the model. To account for the split-plot nature of the study design, we assigned a unique identity number to the twelve experiments and we added this ‘identity number’ as a categorical, random factor to the model to allow for a different intercept for each of the experiments.

We implemented the resulting logistic fixed and mixed effects models in R using the ‘glm’ function for generalized linear effects models and the ‘lmer’ function for generalized linear mixed effects models, respectively. Because the five-factorial model indicated clear-cut differences in treatment effects for the three study strains, we present results of the corresponding four-factorial models for each of the three study strains separately. As the experimental factors were approximately orthogonal, we focused on full models only and did not attempt model reduction or model averaging. We evaluated the performance of all models thoroughly. Because we observed some structure in the model residuals we repeated the analyses including in the model various combinations of (centered) squared model terms; we compared the performance of these model alternatives in terms of their AIC-values (an Information-Theoretic measure of model performance; [37]); results of this comparison are presented in Appendix S4.

## Results

### Leptinotarsa decemlineata – effects of male density and female density

**Figure 4:**
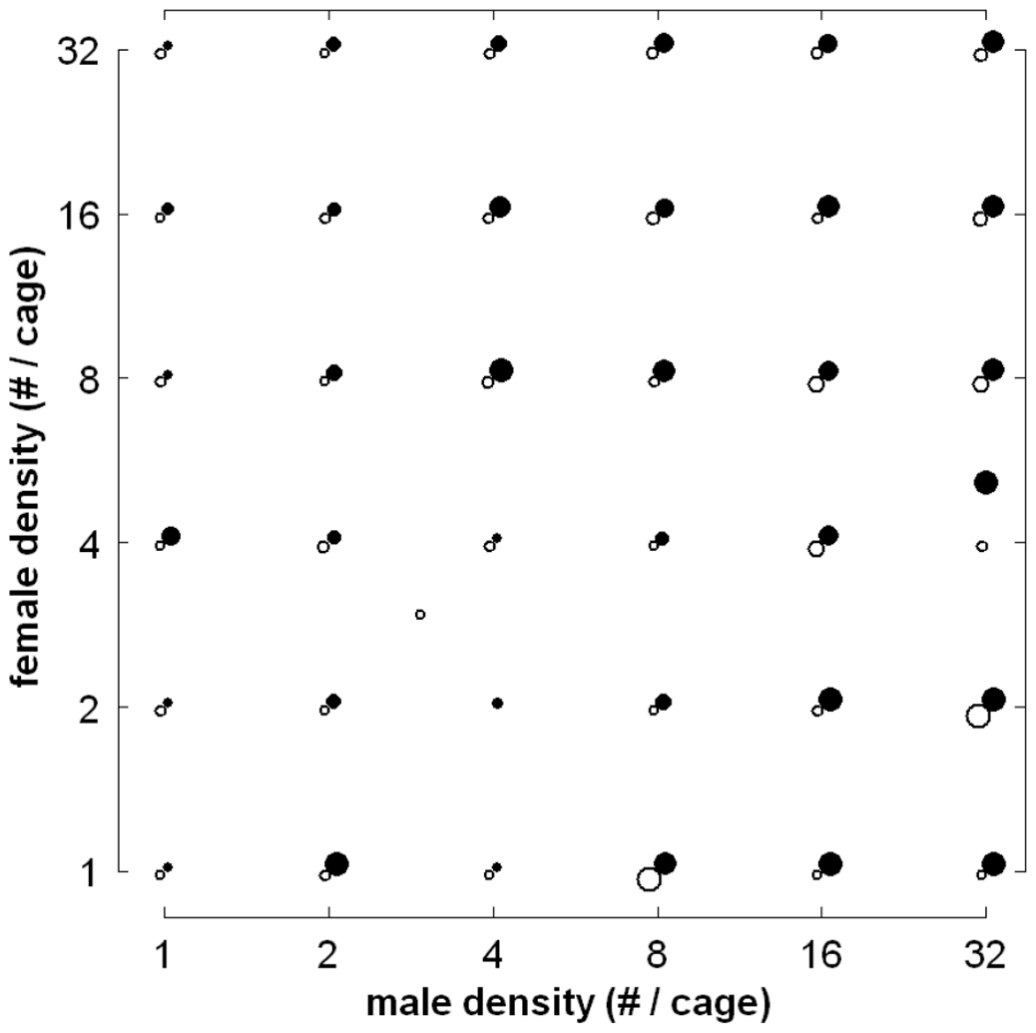
Proportion of female *Leptinotarsa decemlineata* fertilized at different combinations of male density and female density. Open and filled symbols depict single observations from short (3 h) trials and long (22 h) trials, respectively. Symbol size indicates the proportion of females fertilized, with the smallest and largest symbols corresponding to none and all of the females being fertilized, respectively. Long Tick marks indicate treatment levels. For representational purposes, results of short trials and long trials have been slightly shifted diagonally. Note that in two trials (short M_4_,F_2_ and long M_32_,F_4_), treatment levels were not as intended; dissection proved one supposed male to be a female (see Appendix S3).

The proportion of females fertilized varied considerably across replicates, covering the full range in both short and long trials (see Appendix S4 for the full data set). Visual inspection of the results (Fig. 4) suggests several effects of the treatment factors. Most notably, the proportion of females fertilized appears to increase with male density and to be higher in long trials than in short trials.

Logistic regression (Table 1; for a graphical representation and for interpretation of the various test statistics presented, see Appendix S4) confirms this suggestion, showing that, qualitatively, both trial duration and male density had a strong, positive effect on the ln-transformed odds of fertilization (i.e. the ln-transformed ratio of the probability of fertilization over the probability of no fertilization), whereas there was no indication of effects of female density, of interaction terms, and of the block factors. Quantitatively, doubling male density led to a 102% increase of the ln-transformed odds of fertilization of a female, while doubling female density implied but a 6% decrease of these odds (Table 1). Assuming effects of trial duration to be linear, the ln-transformed odds of fertilization increased 15% per hour (Table 1), so that the net effect of trial duration was pronounced: increasing the duration of trials with 19 h led to a 1280% increase of the ln-transformed odds of fertilization.

**Table 1:** Results of the (logistic) regression model of the ln-transformed odds of fertilization for female *Leptinotarsa decemlineata* (Colorado potato beetles)†.

n = 72, AIC = 131.59 Treatment effects (fixed)	β ± s.e.	95% CI (low, high)	odds ratio	z-value
**whole plot**				
constant	**−0.80±0.22**	(−1.23, −0.36)	-	-
trial duration ‘TD’	**0.14±0.02**	(0.09, 0.18)	1.15	5.95
**subplot**				
log_2_(male density) ‘M’	**0.71±0.11**	(0.48, 0.93)	2.02	6.17
log_2_(female density) ‘F’	−0.06±0.10	(−0.25, 0.13)	0.94	−0.63
TD·M	0.01±0.01	(−0.02, 0.03)	1.01	0.49
TD·F	−0.00±0.01	(−0.02, 0.02)	1.00	−0.11
M·F	−0.01±0.06	(−0.13, 0.11)	0.99	−0.13
TD·M·F	0.00±0.01	(−0.01, 0.02)	1.00	0.57

† Parameter estimates ‘β’ and their standard error ‘s.e.’ were computed using the ‘lmer’ function in R. Confidence intervals ‘CI’ of parameter estimates were computed as β ± z_α,2_ · se(β), the ‘odds ratio’, that is, the ratio of the odds of fertilization at two treatment values that differ exactly one unit, was computed as exp(β), and the ‘z-values’ were computed as β/se(β), all conform Collett [38]. Effects that are substantial relative to their standard error are presented in bold to guide the eye. Random effects (σ^2^) of the block factors experimental day (nested within TD) and cage were 0.13 and 0.00, respectively.

**Figure 5:**
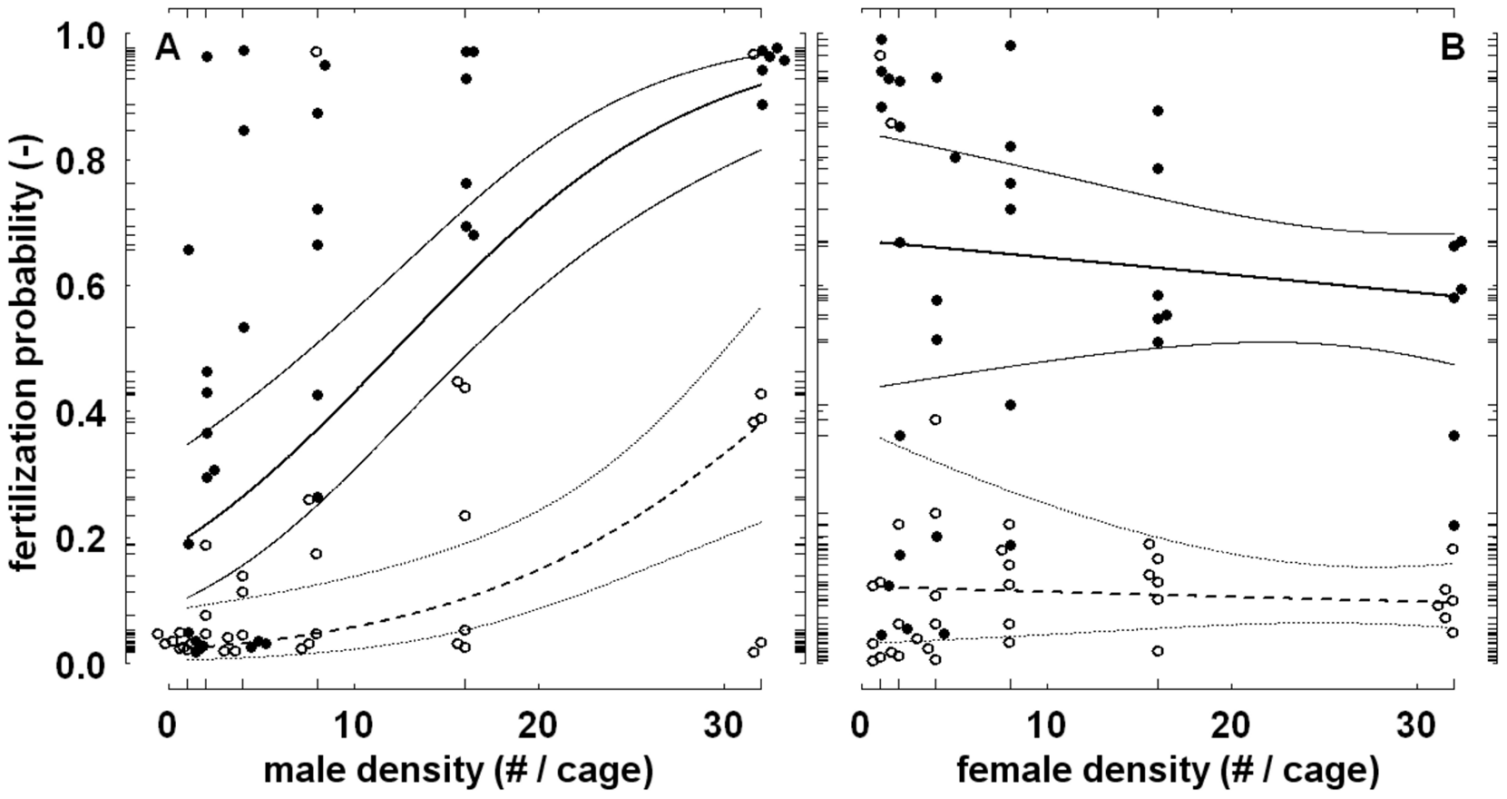
Fertilization probability in relation to male density (panel A) and female density (panel B). Presented are the back-transformed values of the ln-transformed odds (i.e. logits) of fertilization of a female *Leptinotarsa decemlineata*. To avoid taking the natural logarithm of 0, the smallest proportion of fertilized or unfertilized females observed (i.e. 0.03) was added and subtracted to observations of 0 and 1, respectively. Open and filled symbols depict single observations from short trials and long trials, respectively, after variation accounted for by model terms other than the ones depicted in the panel at issue has been taken out. Superimposed are the back-transformed fitted linear regression lines based only on the model terms depicted in the panel at issue (thick lines), with dotted lines and solid lines presenting predictions for short trials and long trials, respectively. The back-transformed approximate 95% confidence intervals (Collett [38] §3.15) of the fitted models are indicated by thin lines. Long tick marks indicate treatment levels and response values. Bracketed information in the axis labels concerns the dimension of the variable at issue (‘−’ indicating dimensionless). For representational purposes, results of short trials and long trials, as well as results overlapping within trial duration, have been slightly shifted horizontally. Note that despite distortion, not all observations are visible at the lower densities. Also note the log-scale of the x-axis.

The corresponding effect of male density on the untransformed female probability of fertilization was non-linear and depended on trial duration (Fig. 5). In short trials, doubling male density increased this probability especially when male density was high, whereas doubling male density in long trials had the strongest effect when few males were present. In short trials, the estimated fertilization probability never exceeded 0.5, regardless of the number of males. In long trials the estimated probability was generally well below 0.5 when only one or two males were present, but generally well above 0.5 in the presence of eight males or more, and close to 1 with 32 males on the plants. Although the observed variation around these estimations was substantial, the probability of fertilization did not vary systematically with female density (Fig. 5).

### Drosophila – Wallace [3]

**Figure 6:**
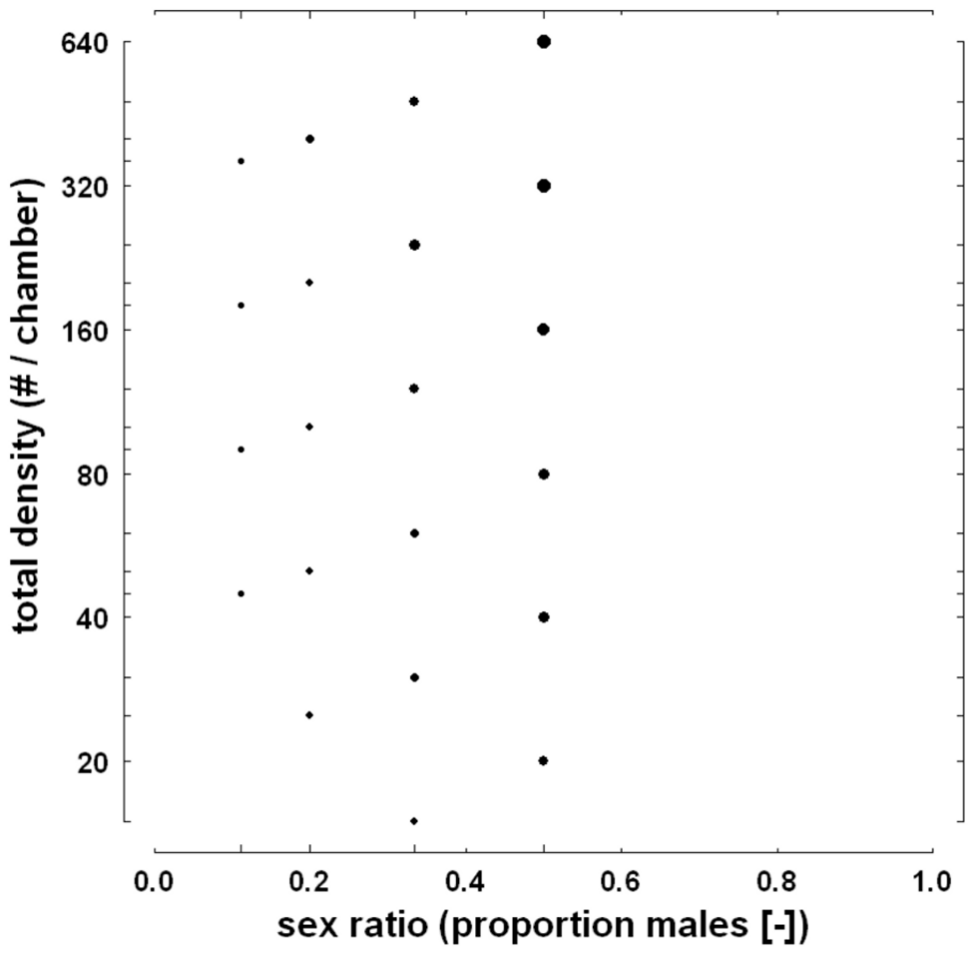
The proportion of female *Drosophila* fertilized at different combinations of sex ratio and total density. Data is from the experiment presented in Wallace [3]. Composition is as in Fig. 4.

**Figure 7:**
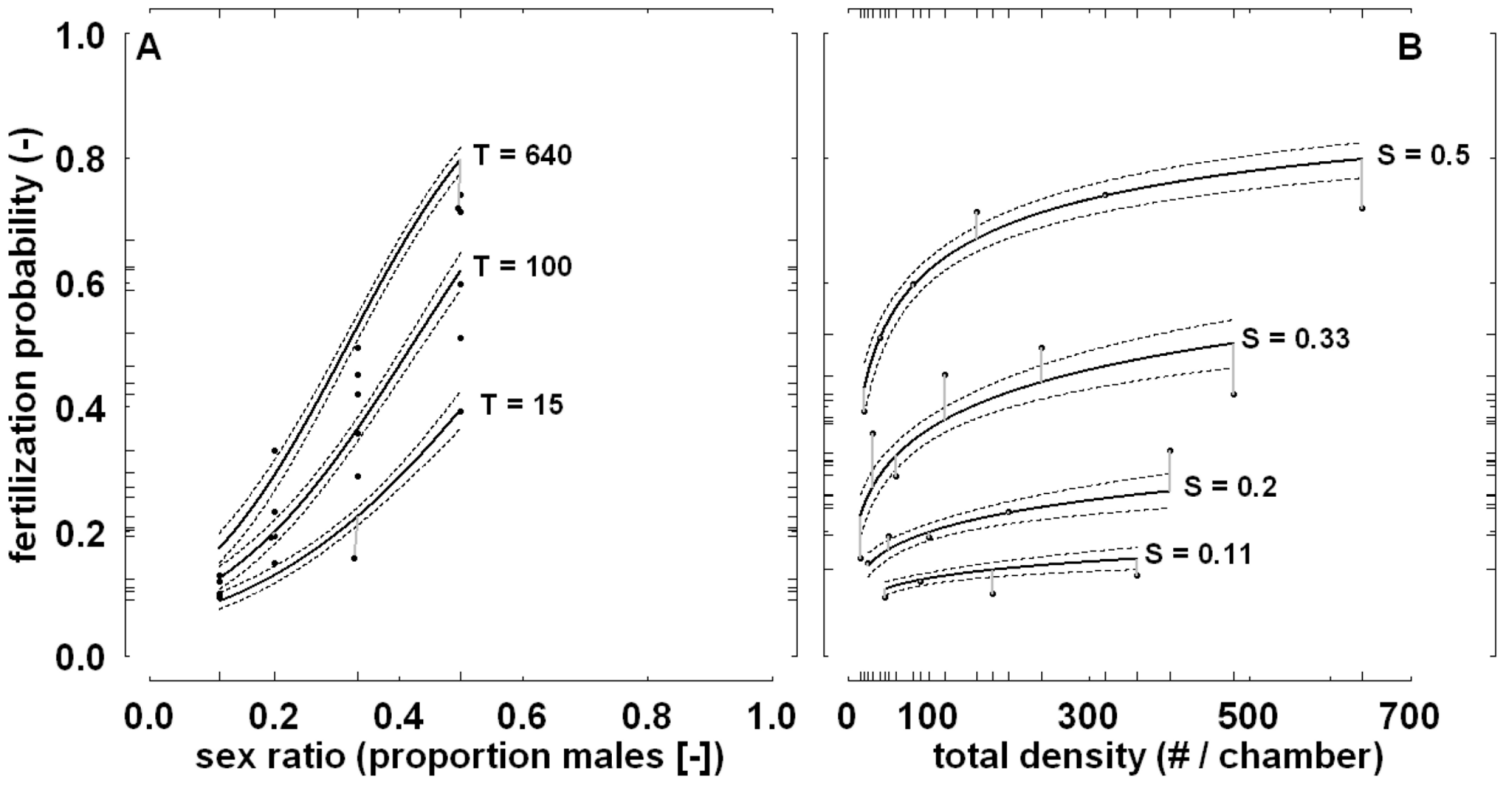
Fertilization probability in relation to sex ratio (panel A) and total density (panel B). Data concerns female *Drosophila* and is from the experiment presented in Wallace [3]. Presented are back-transformed values. Superimposed are the back-transformed fitted linear regression lines (continuous lines) and 95% confidence intervals (dotted lines) calculated at specific levels of total density ‘T’ or sex ratio ‘S’. Grey lines show residuals associated with the depicted regression lines (for representational purposes, corresponding observations have been slightly shifted horizontally in panel A). Long tick marks indicate treatment levels and response values. Note the log-scale of the x-axis in panel B.

The proportion of female *Drosophila* that was fertilized in the experiment presented in Wallace [3] varied considerably, ranging from 0.09 in a trial with sex ratio 1/9 to 0.74 in a trial with sex ratio 1/2. Visual inspection of the results (Fig. 6) suggests that fertilization was positively related to sex ratio, total density, and possibly their combination. Logistic (first-order) regression confirms this suggestion, showing strong, positive main effects of total density and sex ratio on the ln-transformed odds of fertilization (Table 2). It also shows that the joint effect of sex ratio and total density was positive, but moderate (Table 2). Over the range of measurement, the untransformed fertilization probability was strongly, and almost linearly, affected by sex ratio, with a higher probability of fertilization at the less female-biased sex ratios (Fig. 7). The main effect of total density on the fertilization probability was non-linear (Fig. 7); this probability increased especially rapidly when total density was low. Comparison of AIC-values of second-order models including either total density squared (AIC-value: 138.77), sex ratio squared (144.94), both terms squared (137.23), or neither of the two terms squared (145.02), indicated that the predictive ability improved slightly with a quadratic effect of total density, but not sex ratio.

**Table 2:** Results of the (logistic) regression model of the ln-transformed odds of fertilization for female *Drosophila* (fruit flies) in the experiment of Wallace [3] †.

n≥103, AIC = 145.02 Treatment effects (fixed)	β ± s.e.	95% CI (low, high)	odds ratio	z-value
constant	**−0.71±0.04**	(−0.80, −0.63)	-	-
log_2_(total density) ‘T’	**0.24±0.03**	(0.18, 0.30)	1.27	7.82
sex ratio ‘S’	**6.29±0.31**	(5.70, 6.90)	540.27	20.03
T · S	**0.49±0.23**	(0.04, 0.94)	1.63	2.11

† Parameter estimates ‘β’ and their standard error ‘s.e.’ were computed using the ‘glm’ function in R. For details of the computation and interpretation of the other statistics presented, see Table 1.

### Drosophila – Wallace [18]

**Figure 8:**
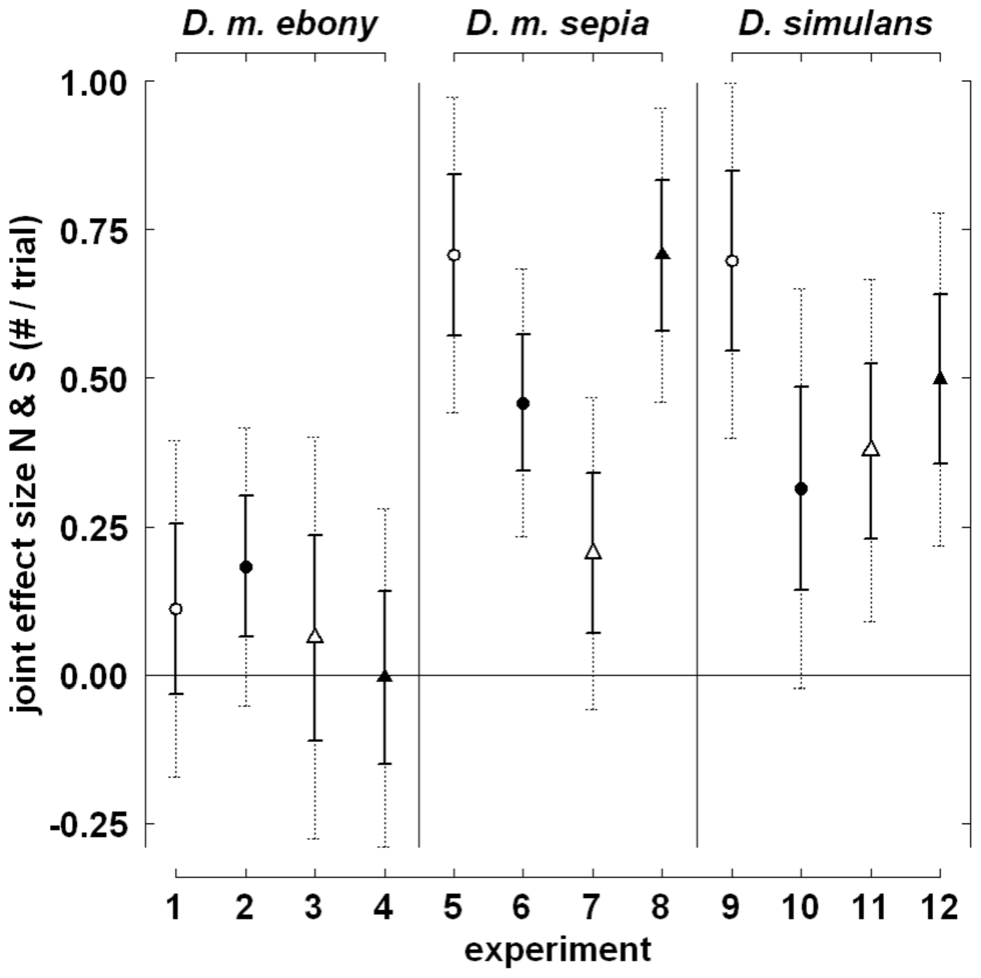
Interaction effects on the ln-transformed odds (i.e. logits) of fertilization of female *Drosophila.* Depicted are the parameter estimates of the joint effect size of the total number of flies (N) and sex ratio (S); data is from the twelve experiments presented in Wallace [18]. Symbols indicate trial duration (30 min: open symbols, 60 min: filled symbols), and the number of mating chambers in the experiment (1: circles, 2: triangles). Solid and dotted error bars indicate the standard error and the 95% confidence intervals of these parameter estimates, respectively. Positive joint effect sizes can be interpreted as indicating that the positive effect of the total number of flies was more pronounced when sex ratio was higher (more male-biased), and vice versa.

Separately, the twelve logistic fixed effects regression analyses of the experiments presented in Wallace [18] show effects of the total number of flies and sex ratio very similar to those observed in our reanalysis of data from Wallace [3]. Females were invariably more likely to be fertilized when the total number of flies was higher (range of estimated main effects on the ln-transformed odds of fertilization: 0.19 to 0.46) and when the proportion of males was higher (3.22 to 6.30). In most experiments with *D. m. sepia* and *D. simulans*, but not in those with *D. m. ebony*, effects of the total number of flies and sex ratio additionally interacted (Fig. 8), as indicated by moderately positive effect sizes, their standard error and the associated 95% confidence interval.

The four-factorial logistic mixed effects regression analysis (Table 3) additionally shows that the joint effect of the total number of flies and sex ratio in *D. m. sepia* and *D. simulans* was not strongly dependent on trial duration, the number of mating chambers, or the combination thereof (Table 3). Likewise, it shows that the ln-transformed odds of fertilization increased with trial duration; for all three study strains, the main effects of the total number of flies and of sex ratio, however, were robust against doubling of trial duration (Table 3). The presence of a second mating chamber lowered the ln-transformed odds of fertilization for *D. m. sepia* or *D. simulans* both directly and by interacting with the effect of sex ratio (Table 3). Variation due to the block factors day and cage was negligible.

**Table 3:** Results of the (logistic) regression model of the ln-transformed odds of fertilization for female of three *Drosophila* strains in the experiments of Wallace [18] †.

	*D. m. ebony* (n≥1164)	*D. m. sepia* (n≥1117)	*D. simulans* (n≥1006)
**Treatment effects (fixed)**			
**whole plot**			
constant	**−1.42±0.02**	**−0.50±0.01**	**−1.28±0.02**
number of chambers ‘nC’	−0.05±0.03	**−0.31±0.03**	**−0.27±0.03**
trial duration ‘TD’	**0.02±0.00**	**0.03±0.00**	**0.03±0.00**
nC·TD	0.01±0.00	−0.01±0.00	−0.02±0.00
**subplot**			
log_2_(total number of flies) ‘N’	**0.36±0.01**	**0.22±0.01**	**0.31±0.01**
sex ratio ‘S’	**3.94±0.11**	**5.38±0.09**	**4.03±0.11**
N·S	0.09±0.07	**0.52±0.06**	**0.47±0.08**
nC·N	0.06±0.02	0.04±0.02	0.03±0.02
nC·S	−0.27±0.21	**−0.73±0.19**	**−0.81±0.22**
TD·N	0.00±0.00	−0.00±0.00	0.00±0.00
TD·S	0.02±0.01	0.03±0.01	0.01±0.01
nC·N·S	−0.12±0.15	−0.13±0.13	−0.07±0.15
TD·N·S	0.00±0.00	0.00±0.00	−0.00±0.01
nC·TD·N	0.00±0.00	−0.00±0.00	0.00±0.00
nC·TD·S	0.04±0.01	−0.02±0.01	−0.04±0.01
nC·TD·N·S	−0.00±0.01	0.03±0.01	0.02±0.01
**Block effect (random)**			
experiment ‘Exp_ID’	0.00	0.00	0.00

† Presented values indicate parameter estimates and their standard error for the treatment factors, and variance for the block factor. Substantial effects with a relatively low standard error are presented in bold to guide the eye.

## Discussion

Our results bear out the notion that understanding and predicting female fertilization probability may require information not just about total density, but also about sex-specific densities. In our experiment with *Leptinotarsa decemlineata*, this is indicated by markedly different effects of male and female density. Fertilization probability increased logistically with male density but was unaffected by female density. The same principle is reflected in all *Drosophila* experiments: fertilization probability increased with the total number of flies and with the proportion of males. For two of the three strains (*D. m. sepia* and *D. simulans*), effects of total density were furthermore apparent in that fertilization probability was lower when a second mating chamber was available.

The multifactorial design of the experiments presented above allows for additional inference regarding the generality of these main effects. In our experiment with *Leptinotarsa decemlineata*, main effects of male density and female density were robust to variation in the density of the other sex and in the duration of trials. In experiments with *Drosophila*, main effects of sex ratio and total density (either varied through the total number of flies or through the number of mating chambers) were likewise robust to variation in trial duration. In experiments with *D. m. ebony*, effects of sex ratio and total density acted independently, whereas in experiments with *D. m. sepia* and *D. simulans* the positive effects of sex ratio were more pronounced when total density was higher (and vice versa).

Together, the main effects and the interaction effects quantitatively describe female per capita fertilization response surfaces. To us, this experimental description reflects the prime value of our analyses. We share the view of Peckarsky [40] that experiments can be of value not only as tests of explicit hypotheses but also as phenomenological descriptions. By minimizing correlations among treatment factors, correlations of treatment factors with uncontrolled variables, and feedback effects of responses on treatment factors, our experimentally determined response surfaces capture the direct effects of the density and the frequency of the two sexes. Sufficiently replicated across species, focus on these direct effects may facilitate both the comparison of response surfaces between-species and the linking of response surfaces to mechanism-based theoretical models of sex-specific, density-dependent reproduction (for examples, see [26], [41]).

### Comparison with previous experimental studies

Few studies have explicitly considered interaction effects on female fertilization probability [16], [22], [42], [43]. Contrary to us, these studies found no interaction effects of sex ratio and total density. The scarcity of studies makes generalization of (the absence of) interaction effects premature, and implies that reported main effects of the density and the frequency of the sexes should be interpreted with care. A therefore tentative look at the results of previous experiments (see Appendix S1) suggests that our findings are qualitatively in line with many previous findings. Positive main effects of male density, over at least part of the ranges examined, have been found in roughly half of about 50 experiments. Similarly, over at least part of the range examined, half of about 50 experiments found no effect of female density, and about 60% of 75 previous studies found positive main effects of sex ratio. Note, though, that there is also a substantial number of studies that (unlike us) found no effect of male density and sex ratio, or a negative effect of female density over at least part of the range examined.

### Relevance

In the *Leptinotarsa decemlineata* experiment, the density and frequency of males and females ranged beyond what is on average found in (experimentally uncontrolled) field conditions (e.g. [44]–[47]); presumably the same holds true for the *Drosophila* experiments. Our treatment levels may still bear relevance to natural aggregations, because sex ratios and densities can be highly variable locally (see Appendix S2). Also, low densities and potentially skewed sex ratios can be expected when new habitat is colonized (e.g. [48]). In case of *Leptinotarsa decemlineata*, high transient densities can furthermore be expected when summer generation beetles emerge en masse on chemically untreated potato fields (max: ≈500 adult beetles per plant [Vahl and Boiteau, personal observation]). In addition, studies with atypical conditions may help to understand why some sex ratios and densities rarely occur and to identify conditions under which fertilization of all females should not be expected – a situation that is perhaps surprisingly common in natural populations of insects [49]–[4951].

Inferences about uncontrolled systems from our findings on experimentally controlled systems should only be drawn with care because the strains used in the *Drosophila* experiments had been in the laboratory for many years [3], [18], because in each of the experiments environmental conditions and characteristics of the study subjects were either standardized or randomized. Care should also be taken in generalizing from our measurements. While fertilization is a prerequisite for successful (sexual) reproduction, there is obviously more to reproduction, and other facets of reproduction may well be affected differentially [1]. Likewise, scaling up to long term, large scale effects can be expected to be far from straightforward [52].

That said, our experimental description of female per capita fertilization response surfaces sets the stage for empirical excursions into mechanisms underlying fertilization dynamics. Although neither our experiment nor those of Wallace [3], [18] were designed specifically to reveal mechanisms, they provide some important clues. Fertilization of female *Leptinotarsa decemlineata* was limited by male density, and not affected by female density. This implies that females can, at least temporarily, be sperm-limited, while simultaneously males had not run out of sperm, as indicated by their ability to fertilize more females when more were present. What limited the number of fertilizations per trial was probably not mating duration either, because copulation and mate guarding takes on average only about 15 minutes in *Leptinotarsa decemlineata* [32], [53]. Rather, a plausible and parsimonious explanation sufficient to explain the observed effects is provided by the idea that searching for mates is not infinitely efficient; if searching for mates takes time, not all females will become fertilized during a finite period of time. The same mechanism may explain the positive effect of sex ratio on the fertilization probability of female *Drosophila*, though this may also have come about through a reduction of competition amongst females at the more male-biased sex ratios.

Although further experimentation is clearly desirable, our analyses also allow for some initial inferences regarding the extent to which effects of sex-specific densities on fertilization probability generalize across populations and species. Our reanalyzes of data from Wallace [3], [18] indicate that treatment effects of sex ratio and the total number of flies on the fertilization success of *D. m. sepia* are quantitatively consistent across studies. Reanalysis of the data from Wallace [18] furthermore shows that treatment effects of sex ratio and total density can quantitatively and qualitative be more comparable between strains of different species (*D. m. sepia*, *D. simulans*) than between strains of the same species (*D. m. ebony*, *D. m. sepia*). Analysis of the effects of sex ratio and total density on the fertilization probability of female *Leptinotarsa decemlineata* (see Appendix S4) shows that treatment effects can also be qualitatively similar in species of different orders (*L. decemlineata* vs. *D. m. sepia* and *D. simulans*).

Accurate estimation of fertilization response surfaces also paves the way for a quantitatively detailed evaluation of relevant theoretical work. Numerous mathematical models have been proposed to capture the relationship between sex-specific densities and reproduction (for partial overviews, see [41], [54]). Depending on the facet of reproduction that is assumed to depend on the density of either sex, these models are known as marriage, mating, fertility, or birth functions [54]. Examination of the relative ability of such functions to describe empirical measurements is rare (but see for instance [24], [26]), perhaps mainly because of the scarcity of rigorous experiments [26], [54]. The short temporal scale of our experiments makes them especially suited for confrontation with models that treat time as a continuous variable (e.g. [41]).

## Conclusions

In summary, male and female density affected the per capita fertilization of female *Leptinotarsa decemlineata* differentially, and fertilization of *Leptinotarsa decemlineata* and *Drosophila* depended on both total density and sex ratio. These findings strengthen experimental evidence for the relevance of sex-specific densities to understand and predict female fertilization probability. The multifactorial, experimental approach exemplified in this study adds a level of quantitative rigor to the study of sex-specific effects of density on reproduction that, if sufficiently replicated across species, should facilitate comparison across species and confrontation of theory with data.

## Acknowledgments

We are grateful to the personnel of the Potato Research Center; in particular we thank Alex, Jean, Kelsey and Kylee for help in preparing the experiment, and Sylvia Holder and John McDonald for their work in the greenhouses. We thank Bob O’Hara for advice on various statistical issues. Members of LEED provided a stimulating working environment. We are especially grateful to Maria del Mar Delgado for her extensive contribution to the composition of the text.

## Supporting Information

Appendix S1 **Overview of previous experiments.**
(DOCX)

Appendix S2 **Field observations of sex-specific densities of** ***Leptinotarsa decemlineata*** **.**
(DOCX)

Appendix S3 **Supplementary methods** ***Leptinotarsa decemlineata*** **experiment.**
(DOCX)

Appendix S4 **Supplementary results** ***Leptinotarsa decemlineata*** **and** ***Drosophila*** **experiments.**
(DOCX)
