# Supplementary material for: Female Fertilization: Effects of Sex-Specific Density and Sex Ratio Determined Experimentally for Colorado Potato Beetles and Drosophila Fruit Flies
Source: PLoS One. 2013 Apr 12;8(4):e60381. doi: 10.1371/journal.pone.0060381 (PMC3625176; doi:10.1371/journal.pone.0060381)
Supplement: Appendix S1 — Overview of previous experiments. (DOCX) [file pone.0060381.s001.docx]

# Appendix S1: overview of previous experiments

**Main paper**: Vahl et al. 2013. **Female fertilization: effects of sex-specific density and sex ratio determined experimentally for Colorado potato beetles and *Drosophila* fruit flies**.

Effects of the density and frequency of the two sexes on the probability of fertilization of a female have been determined experimentally by numerous studies, together covering a wide range of species (Table S1.1).

**Table S1.1**. Experimentally-determined effects of the (proximate) density or frequency of (adult) males and females on female fertilization probability^†^.

| **Phylum** Class | **Order** | **Species** | **Reference** | **Response^a^** | **M^b^** | **F^b^** | **S^b^** | **Additional factors^c^** |
| --- | --- | --- | --- | --- | --- | --- | --- | --- |
| **Arthropoda** |  |  |  |  |  |  |  |  |
| Insecta | Coleoptera | *Euscepes postfasciatus* | Kumano et al. 2010 [1] | y_1_ | + | na | + | na |
|  |  | *Leptinotarsa decemlineata* | This study | logit (y_2_) | + (12) | 0 (12) | + (2) | x_1_, x_2_ |
|  |  | *Sitophilus oryzae* | Campbell 2005 [2] | arcsine (y_1_) | + | na | + | na |
|  | Diptera | *Anopheles arabiensis* | Verhoek and Takken 1994 [3] | y_2_ | + | na | + | na |
|  |  | *A. gambiae* | Verhoek and Takken 1994 [3] | y_2_ | + | na | + | na |
|  |  |  | Benedict and Rafferty 2002^θ^ [4] | logit (y_2_) | + (4) | na | + (4) | x_3_, x_4_ |
|  |  | *Ceratitis capitata* | Kraaijeveld et al. 2005 [5] | logit (y_1_) | + | na | + | na |
|  |  | *Delia antique* | Robinson 1980 [6] | y_2_ | na | na | 0 (3) | x_5_ |
|  |  | *D. platura* | Hough-Goldstein et al. 1987^θ^ [7] | log (y_3_) | 0, + | -, + | -, 0, + (2)^φ^ | x_6_ |
|  |  | *Drosophila melanogaster* | Jacobs 1960^θ^ [8] | y_1_ | - | na | - | na |
|  |  |  | Søndergaard 1985 [9] | y_3_ | 0 (5), + (7)^φ^ | - (5), 0 (1)^φ^ | 0 (5), + (7)^φ^ | x_4_, x_7_ |
|  |  |  | Søndergaard 1985^θ^ [9] | y_3_ | 0 (3), +^φ^ | 0 (2)^φ^ | 0 (3), +^φ^ | x_7_ |
|  |  |  | Dowse et al. 1986 [10] | y_1_ | + | na | + | na |
|  |  |  | Friberg and Arnqvist 2003 [11] | sqrt (y_1_) | 0 (2) | na | 0 (2) | x_8_ |
|  |  |  | Wigby and Chapman 2004 [12] | log (y_1_) | + | - | + | na |
|  |  |  | Reuter et al. 2008 [13] | logit (y_3_) ^*^ | + | - | + | na |
|  |  |  | This study (data from Wallace [14]) | logit (y_3_) | na | na | + | na |
|  |  |  | This study (data from Wallace [15]) | logit (y_3_) | na | na | + (8) | x_2_, x_9_, x_10_ |
|  |  | *D. persimilis* | Spiess and Spiess 1969^θ^ [16] | y_1_ | + | - | + | na |
|  |  | *D. simulans* | This study (data from Wallace [15]) | logit (y_3_) | na | na | + (4) | x_2_, x_10_ |
|  | Hemiptera | *Oncopeltus fasciatus* | Economopoulos and Gordon 1971^θ^ [17] | y_1_ | + | na | + | na |
|  | Hymenoptera | *Copidosoma floridanum* | Strand 1989 [18] | arcsine (y_2_) | na | - | + | na |
|  |  | *Nasonia vitripennis* | Burton-Chellew et al. 2007^θ^ [19] | y_3_^*^ | + | na | + | na |
|  | Lepidoptera | *Austromusotima camptozonale* | Boughton et al. 2007 [20] | y_2_ | 0, + | na | 0, + | x_5_ |
|  |  | *Corcyra cephalonica* | Etman et al. 1988 [21] | y_2_ | 0 (2)^φ^ | 0 (2)^φ^ | 0 (3)^φ^ | na |
|  |  | *Earias insulana* | Klein et al. 1983 [22] | y_2_ | 0 (2), + (3) | - (3), 0 (2) | 0 (2), + (3) | x_5_, x_10_ |
|  |  | *Epiphyas postvittana* | Danthanarayana and Gu 1991^θ^ [23] | y_2_ | + | - | + (2)^φ^ | na |
|  |  | *Heliothis virescens* | Guerra et al. 1972^θ^ [24] | y_2_ | 0 | - | 0, +^φ^ | na |
|  |  | *H. zea* | Jones et al. 1979^θ^ [25] | y_2_ | + | - | + | na |
|  |  | *Phthorimaea operculella* | Makee and Saour 2001 [26] | arcsine (y_2_) | 0 (7) | na | 0 (7) | x_11_ |
|  |  | *Plodia interpunctella* | Brower 1975^θ^ [27] | y_2_ | 0 | - | 0, +^φ^ | na |
|  |  | *Trichoplusia ni* | Henneberry and Kishaba 1967^θ^ [28] | y_2_ | + | - | + (2)^φ^ | na |
| Malacostraca | Decapoda | *Pacifastacus leniusculus* | Celada et al. 2005^θ^ [29] | y_3_ | 0 | na | 0 | na |
|  |  | *Telmessus cheiragonus* | Kamio et al. 2003^θ^ [30] | y_2_ | 0 | 0 | 0 | na |
|  | Isopoda | *Asellus aquaticus* | Karlsson et al. 2010 [31] | **y_1_** | **0, +** | **-, 0** | **0, +** | **x_9_** |
| **Chordata** |  |  |  |  |  |  |  |  |
| Aves | Anseriformes | *Anas platyrhynchos* | Davis et al. 1993 [32] | arcsine (y_3_) | 0 (2) | 0 (2) | 0 (2) | x_7_ |
|  | Galliformes | *Coturnix coturnix* | Hughes et al. 1980 [33] | y_3_ | na | 0 | 0 | na |
|  |  | *Gallus gallus* | Al-Rawi 1980 [34] | y_3_ | na | 0 | 0 | na |
|  |  | *Phasianus colchicus* | Bates et al. 1987 [35] | arcsine (y_3_) | na | 0 (3), + | -, 0 (3) | x_7_, x_12_ |
| Mammalia | Artiodactyla | *Bos spp.* | Neville et al. 1979 [36] | y_3_ | na | 0, + | -, 0 | x_12_ |
|  |  |  | Neville et al. 1988 [37] | y_3_ | na | 0 | 0 | na |
|  |  |  | Pexton et al. 1990 [38] | y_4_ | na | 0 | 0 | na |
|  |  |  | Healy et al. 1993 [39] | y_4_ | + | na | + | na |
|  |  | *Ovis aries* | Lightfoot and Smith 1968 [40] | y_3_ | na | - (3) | + (3) | x_6_ |
|  |  |  | Dawe et al. 1970 [41] | arcsine (y_3_) | 0 (11), + (2) | na | 0 (11), + (2) | x_12_, x_13_ |
|  |  |  | Dawe et al. 1974 [42] | y_3_ | 0 (10), + (7) | na | 0 (10), + (7) | x_11_, x_13_, x_14_ |
|  |  |  | Allison 1975 [43] | y_3_ | na | 0 (2) | 0 (2) | x_12_ |
|  |  |  | Allison 1975 [44] | y_4_ | na | -, 0 (4) | 0 (4), + | x_13_ |
|  |  |  | Bryant and Tomkins 1975 [45] | y_3_ | na | - | + | na |
|  |  |  | Allison and Davis 1976^θ^ [46] | y_3_ | na | - (2), 0 (6) | 0 (6), + (2) | x_12_, x_14_, x_15_ |
|  |  |  | Allison 1977 [47] | y_3_ | na | -, 0 (3) | 0 (3), + | x_6_, x_14_ |
|  | Rodentia | *Clethrionomys glareolus* | Klemme et al. 2007 [48] | y_3_ | 0 | 0 | 0 | na |
|  |  |  | Mills et al. 2007^θ^ [49] | y_3_ | 0 | na | 0 | na |
|  |  | *Microtus ochrogaster* | Hodges et al. 2002 [50] | y_4_ | na | 0 (2)^φ^ | 0 (2)^φ^ | na |
|  |  | *Mus spicilegus* | Gouat and Féron 2005 [51] | y_3_ | na | - | + | na |
| Reptilia | Squamata | *Lacerta vivipara* | Dreiss et al. 2010 [52] | logit (y_3_) | na | na | 0 | na |
| **Nematoda** |  |  |  |  |  |  |  |  |
| Adenophorea | Mermithida | *Octomyomermis muspratti* | Petersen 1978^θ^ [53] | y_5_ | + | na | + | na |
| **Tracheophyta** |  |  |  |  |  |  |  |  |
| Magnoliopsida | Begoniales | *Begonia gracilis* | Castillo et al. 2002 [54] | arcsine (y_6_) | + (2) | - (2) | + (2) | x_16_ |
|  | Campanulales | *Lobelia siphilitica* | Proell 2009 [55] | y_6_ | 0 (2) | 0 (2) | 0 (2) | x_5_ |
|  | Caryophyllales | *Silene vulgaris* | McCauley and Brock 1998 [56] | y_6_^*^ | 0, + | -, 0 | 0, + | x_6_ |
|  | Fabales | *Chamaecrista fasciculata* | Williams and Fenster 1998 [57] | y_6_^*^ | 0 | 0 | 0 | na |
|  | Zygophyllales | *Kallstroemia grandiflora* | Cuevas et al. 2008 [58] | y_6_ | 0 | 0 | 0 | na |

**^†^** We excluded studies written in a language other than English, inter-specific studies, and studies in which data presentation was incomplete, trials were of variable length, variation in density concerned the density of bystanders or sterilized males, no males were present (parthenogenetic species), organisms of one sex were not free to move or reproduce, and organisms were neither spatially confined nor sessile.

^a^ As measures of fertilization probability we accepted the percentage of females in copula ‘y_1_’, inseminated ‘y_2_’, with eggs (laid) or progeny ‘y_3_’, pregnant ‘y_4_’, exhibiting egg development ‘y_5_’, and the percentage of female flowers producing fruit ‘y_6_’. Response variables were either untransformed, or transformed using a ‘logit’-, ‘arcsine’-square-root-, ‘log’-, or square-root ‘sqrt’-transformation.

^b^ Symbols indicate whether the treatment main effects of male density ‘M’, female density ‘F’, and sex ratio ‘S’ were negative ‘-‘ or positive ‘+’ over at least part of the range examined, whether no treatment effects were detected ‘0’, or whether no information was available ‘na’. When a single study presented results for multiple categories of an additional treatment factor, the number of times a particular treatment effect was observed is indicated in brackets; qualitatively different effects per treatment factor are separated by a comma. When treatment effects were measured repeatedly over time, only effects concerning the longest time span are presented. All effects presented are conditional on male density, female density or total density.

^c^ Several factors were included in the designs of the various experiments as additional treatment factors (covariates not included): the density of the other sex ‘x_1_’, trial duration ‘x_2_’, male strain ‘x_3_’, female strain ‘x_4_’, total density ‘x_5_’, replicate ‘x_6_’, light condition ‘x_7_’, male size ‘x_8_’, strain ‘x_9_’, volume ‘x_10_’, female age ‘x_11_’, year ‘x_12_’, location ‘x_13_’, female age composition ‘x_14_’, surface area ‘x_15_’, and flower density ‘x_16_’.

^θ^ The effects presented here were not supported by inferential statistics.

^φ^ Multiple treatment effects were found in this study (partly) because of conditioning on more than one of the main treatment factors (male density, female density and total density).

^*^ Treatment effects in Burton-Chellew et al. [19] concern the percentage of females with diploid offspring. Treatment effects in Reuter et al. [13] are conditional on effective population size rather than on total density. Treatment effects in McCauley and Brock [56] and Williams and Fenster [57] concern the density and frequency of hermaphrodites rather than the density and frequency of males.

**REFERENCES**

1. Kumano N, Kuriwada T, Shiromoto K, Haraguchi D, Kohama T (2010) Effect of body size and sex ratio on male alternative mating tactics of the West Indian sweetpotato weevil, *Euscepes postfasciatus*. Entomol Exp Appl 135: 154-161.
2. Campbell JF (2005) Fitness consequences of multiple mating on female *Sitophilus oryzae* L. (Coleoptera: Curculionidae). Environ Entomol 34: 833-843.
3. Verhoek BA, Takken W (1994) Age effects on the insemination rate of *Anopheles gambiae* s.l. in the laboratory. Entomol Exp Appl 72: 167-172.
4. Benedict MQ, Rafferty CS (2002) Unassisted isolated-pair mating of *Anopheles gambiae* (Diptera: Culicidae) mosquitoes. J Med Entomol 39: 942-944.
5. Kraaijeveld K, Katsoyannos BI, Stavrinides M, Kouloussis NA, Chapman T (2005) Remating in wild females of the Mediterranean fruit fly, *Ceratitis capitata*. Anim Behav 69: 771-776.
6. Robinson AS (1980) Effect of sex ratio at three densities on reproduction in laboratory colonies of *Delia* (= *Hylemya*) *antigua* Meig. Z Angew Entomol 90: 82-89.
7. Hough-Goldstein JA, Hess KA, Cates SM (1987) Group effect on seedcorn maggot (Diptera: Anthomyiidae) mating behavior. Ann Entomol Soc Am 80: 520-523.
8. Jacobs ME (1960) Influence of light on mating of *Drosophila melanogaster*. Ecology 41: 182-188.
9. Søndergaard L (1985) Mating competition in artificial populations of *Drosophila melanogaster* polymorphic for ebony. 1. Role of light and the male to female ratio on mating success. Hereditas 103: 47-55.
10. Dowse HB, Ringo JM, Barton KM (1986) A model describing the kinetics of mating in Drosophila. J Theor Biol 121: 173-183.
11. Friberg U, Arnqvist G (2003) Fitness effects of female mate choice: preferred males are detrimental for *Drosophila melanogaster* females. J Evolution Biol 16: 797-811.
12. Wigby S, Chapman T (2004) Female resistance to male harm evolves in response to manipulation of sexual conflict. Evolution 58: 1028-1037.
13. Reuter M, Linklater JR, Lehmann L, Fowler K, Chapman T, et al. (2008) Adaptation to experimental alterations of the operational sex ratio in populations of *Drosophila melanogaster*. Evolution 62: 401-412.
14. Wallace B (1985) Mating kinetics in *Drosophila*. Behav Sci 30: 149-154.
15. Wallace B (1990) Male male interactions and mating kinetics in *Drosophila*. Behav Genet 20: 405-421.
16. Spiess EB, Spiess LD (1969) Mating propensity, chromosomal polymorphism, and dependent conditions in *Drosophila persimilis*. 2. Factors between larvae and between adults. Evolution 23: 225-236.
17. Economopoulos AP, Gordon HT (1971) Chemosterilization of *Oncopeltus fasciatus*.1. Control experiments. J Econ Entomol 64: 1351-1354.
18. Strand MR (1989) Clutch size, sex ratio and mating by the Polyembryonic encyrtid *Copidosoma floridanum* (Hymenoptera, Encyrtidae). Fla Entomol 72: 32-42.
19. Burton-Chellew MN, Sykes EM, Patterson S, Shuker DM, West SA (2007) The cost of mating and the relationship between body size and fitness in males of the parasitoid wasp *Nasonia vitripennis*. Evol Ecol Res 9: 921-934.
20. Boughton AJ, Wu J, Pemberton RW (2007) Mating biology of *Austromusotima camptozonale* (Lepidoptera : Crambidae), a potential biological control agent of Old World climbing fern, *Lygodium microphyllum* (Schizaeaceae). Fla Entomol 90: 509-517.
21. Etman AAM, El-Sayed FMA, Eesa NM, Moursy LE (1988) Laboratory studies on the development, survival, mating behaviour and reproductive capacity of the rice moth, *Corcyra cephalonica* (Stainton)(Lepidoptera, Galleriidae). J Appl Entomol 106: 232-240.
22. Klein M, Yathom S, Keren S, Levski S, Tal S (1983) The effects of population density and sex ratio frequency of *Earias insulana* males and females in laboratory cultures. Phytoparasitica 11: 145-149.
23. Danthanarayana W, Gu H (1991) Multiple mating and its effect on the reproductive success of female *Epiphyas postvittana* (Lepidoptera: Tortricidae). Ecol Entomol 16: 169-175.
24. Guerra AA, Wolfenbarger DA, Garcia RD (1972) Factors affecting reproduction of Tobacco budworm in the laboratory. J Econ Entomol 65: 1341-1343.
25. Jones RL, Perkins WD, Sparks AN (1979) Effect of sex ratios on reproduction by the corn earworm in the laboratory. Ann Entomol Soc Am 72: 35-37.
26. Makee H, Saour G (2001) Factors influencing mating success, mating frequency, and fecundity in *Phthorimaea operculella* (Lepidoptera: Gelechiidae). Environ Entomol 30: 31-36.
27. Brower JH (1975) *Plodia interpunctella*: effect of sex ratio on reproductivity. Ann Entomol Soc Am 68: 847-851.
28. Henneberry TJ, Kishaba AN (1967) Mating and oviposition of cabbage looper in laboratory. J Econ Entomol 60: 692-696.
29. Celada JD, Antolin JI, Carral JM, Saez-Royuela M, Rodriguez R (2005) Successful sex ratio of 1M : 4F in the astacid crayfish *Pacifastacus leniusculus* Dana under captive breeding conditions. Aquaculture 244: 89-95.
30. Kamio M, Matsunaga S, Fusetani N (2003) Observation on the mating behaviour of the helmet crab *Telmessus cheiragonus* (Brachyura: Cheiragonidae). J Mar Biol Assoc UK 83: 1007-1013.
31. Karlsson K, Eroukhmanoff F, Svensson EI (2010) Phenotypic plasticity in response to the social environment: effects of density and sex ratio on mating behaviour following ecotype divergence. PLOS One 5: 6.
32. Davis GS, Parkhurst CR, Brake J (1993) Light intensity and sex ratio effects on egg production, egg quality characteristics and fertility in breeder Pekin ducks. Poultry Sci 72: 23-29.
33. Hughes BL, Jones JE, Resseguie WD (1980) Effect of male to female ratios on reproduction of caged Coturnix D1 breeders. Poultry Sci 59: 1339-1341.
34. Al-Rawi BA (1980) Sex ratio effects on egg production. Poultry Sci 59: 1546-1547.
35. Bates DP, Hanson LE, Cook ME, Wentworth BC, Sunde ML, et al. (1987) Lighting and sex-ratio for breeding ringnecked pheasants in confined housing. Poultry Sci 66: 605-612.
36. Neville WE, Smith JB, McCormick WC (1979) Reproductive performance of 2-year old and 3-year old bulls assigned 25 or 40 cows during the breeding period. J Anim Sci 48: 1020-1025.
37. Neville WE, Richardson KL, Utley PR (1988) Breeding performance of bulls assigned to 40 or 50 cows per bull during the breeding period. J Anim Sci 66: 613-617.
38. Pexton JE, Farin PW, Rupp GP, Chenoweth PJ (1990) Factors affecting mating activity and pregnancy rates with beef bulls mated to estrus synchronized females. Theriogenology 34: 1059-1070.
39. Healy VM, Boyd GW, Gutierrez PH, Mortimer RG, Piotrowski JR (1993) Investigating optimal bull-heifer ratios required for estrus-synchronized heifers. J Anim Sci 71: 291-297.
40. Lightfoot RJ, Smith JAC (1968) Studies on the number of ewes joined per ram for flock matings under paddock conditions. 1. Mating behaviour and fertility. Aust J Agr Res 19: 1029-1042.
41. Dawe ST, Bennett NW, Donnelly FB, Ferguson BD, Rive JP, et al. (1970) The comparative reproductive performance of ewes joined to one or three per cent of rams. Proc Aust Soc Anim Prod 8: 317-320.
42. Dawe ST, Archer WR, Bennett NW, Brunskill A, Cahill JR, et al. (1974) The effect of ram percentage on the fertility of maiden ewes. Proc Aust Soc Anim Prod 10: 274-278.
43. Allison AJ (1975) Flock mating in sheep. 1. Effect of number of ewes joined per ram on mating behavior and fertility. New Zeal J Agr Res 18: 1-8.
44. Allison AJ (1975) Ewe and ram fertility in commercial flocks mated with different numbers of ewes per ram. New Zeal J Exp Agr 3: 161-167.
45. Bryant MJ, Tomkins T (1975) The flock mating of progestagen-synchronized ewes. 1. The influence of ram-to-ewe ratio upon mating behaviour and lambing performance. Anim Prod 20: 381-390.
46. Allison AJ, Davis GH (1976) Studies of mating behaviour and fertility of Merino ewes. 1. Effects of number of ewes joined per ram, age of ewe, and paddock size. New Zeal J Exp Agr 4: 259-267.
47. Allison AJ (1977) Flock mating in sheep. 2. Effect of number of ewes per ram on mating behavior and fertility of 2-tooth and mixed age Romney ewes run together. New Zeal J Agr Res 20: 123-128.
48. Klemme I, Ylönen H, Eccard JA (2007) Reproductive success of male bank voles (*Clethrionomys glareolus*): the effect of operational sex ratio and body size. Behav Ecol Sociobiol 61: 1911-1918.
49. Mills SC, Grapputo A, Koskela E, Mappes T (2007) Quantitative measure of sexual selection with respect to the operational sex ratio: a comparison of selection indices. Proc Roy Soc B – Biol Sci 274: 143-150.
50. Hodges KE, Mech S, Wolff JO (2002) Sex and the single vole: Effects of social grouping on prairie vole reproductive success. Ethol 108: 871-884.
51. Gouat P, Féron C (2005) Deficit in reproduction in polygynously mated females of the monogamous mound-building mouse *Mus spicilegus*. Reprod Fert Develop 17: 617-623.
52. Dreiss AN, Cote J, Richard M, Federici P, Clobert J (2010) Age- and sex-specific response to population density and sex ratio. Behav Ecol 21: 356-364.
53. Petersen JJ (1978) Effects of male-female ratios on mating and egg production in *Octomyomermis muspratti* (Mermithidae: Nematoda). J Invertebr Pathol 31: 103-105.
54. Castillo RA, Cordero C, Dominguez CA (2002) Are reward polymorphisms subject to frequency- and density-dependent selection? Evidence from a monoecious species pollinated by deceit. J Evolution Biol 15: 544-552.
55. Proell J (2009) Population sex ratio and size affect pollination, reproductive success, and seed germination in gynodioecious *Lobelia siphilitica*: evidence using experimental populations and microsatellite genotypes: M. Sc. Thesis, Kent State University (UMI Microform 1468476). Ann Arbor: ProQuest LLC. 96 p.
56. McCauley DE, Brock MT (1998) Frequency-dependent fitness in *Silene vulgaris*, a gynodioecious plant. Evolution 52: 30-36.
57. Williams HL, Fenster CB (1998) Ecological and genetic factors contributing to the low frequency of male sterility in *Chamaecrista fasciculata* (Fabaceae). Am J Bot 85: 1243-1250.
58. Cuevas E, Parker IM, Molina-Freaner F (2008) Variation in sex ratio, morph-specific reproductive ecology and an experimental test of frequency-dependence in the gynodioecious *Kallstroemia grandiflora* (Zygophyllaceae). J Evolution Biol 21: 1117-1124.
