# Supplementary material for: Female Fertilization: Effects of Sex-Specific Density and Sex Ratio Determined Experimentally for Colorado Potato Beetles and Drosophila Fruit Flies
Source: PLoS One. 2013 Apr 12;8(4):e60381. doi: 10.1371/journal.pone.0060381 (PMC3625176; doi:10.1371/journal.pone.0060381)
Supplement: Appendix S2 — Field observations of sex-specific densities of Leptinotarsa decemlineata . (DOCX) [file pone.0060381.s002.docx]

# Appendix S2: Field observations of sex-specific densities of *Leptinotarsa decemlineata*

**Main paper**: Vahl et al. 2013. **Female fertilization: effects of sex-specific density and sex ratio determined experimentally for Colorado potato beetles and *Drosophila* fruit flies**.

# METHODS

From 26 June till 21 August 2008, we made weekly observations of the number of beetles per plant in an insecticide-free potato field of the AAFC Potato Research Center (field ‘r1’). This field was divided in 120 cells; each week one a priori randomly determined plant per cell was scrutinized for the presence of beetles at each visit. In total this yielded 1080 observations of the number of beetles per plant. In the first seven weeks we sexed all beetles that we observed in this survey; beetles were sexed in the field with the use of a 10× magnifying glass on the basis of sex-specific characteristics of the last abdominal segment [1]. This yielded 199 sex-specific observations (641 plants contained no beetles). In the last two weeks (14 and 21 August) beetles became very abundant, and therefore we only sexed beetles on 163 plants that contained 20 beetles or less. As 117 plants contained no beetles, this yielded an extra 46 sex-specific observations. For the other 77 plants observed in the last two weeks, we estimated the total number of beetles without sexing them.

On three dates (17 July, 24 July, 14 August), we additionally counted the number of males and females per plant in a second insecticide-free potato field of the AAFC Potato Research Center (field ‘nursery’). On the first two dates, we systematically counted, sexed and collected all beetles from a united series of plants. This yielded an extra 82 sex-specific observations of the number of beetles per plant (177 plants contained no beetles). On the third date, we scrutinized 107 plants that were selected in an *ad hoc* manner, yielding an extra 63 sex-specific observations. Collected beetles were frozen and their sex was later verified through dissection.

# RESULTS

The 390 sex-specific densities from either field are presented in Figure S2.1. A frequency distribution of all 1447 total per plant densities is presented in Table S2.1, and a frequency distribution of sex ratios on the 390 plants that contained at least one beetle is presented in Table S2.2. Note that because plant size varied considerably over time, absolute densities should be interpreted with care.

# REFERENCES

1. Pelletier Y (1993) A method for sex determination of the Colorado potato beetle pupa, *Leptinotarsa decemlineata* (Coleoptera: Chrysomelidae). Entomol News 104: 140-142.

**Table S2.1**. Total number of *Leptinotarsa decemlineata* (Colorado potato beetles) per plant as observed during nine weeks in two insecticide-free potato fields^†^.

| **total number** | **0** | **1 – 5** | **6 – 10** | **11 – 25** | **26 – 50** | **51 – 100** | **101 – 200** | **201 – 500** |
| --- | --- | --- | --- | --- | --- | --- | --- | --- |
| **frequency** | 980 | 372 | 23 | 22 | 26 | 10 | 11 | 3 |

^†^ ‘Total number’ is the number of beetles per plant, regardless of their sex.

**Table S2.2**. *Leptinotarsa decemlineata* sex ratios per plant as observed during nine weeks in two insecticide-free potato fields^†^.

| **sex ratio** | **0** | **[0 – 1/3]** | **1/3** | **[1/3 – 1/2]** | **0.5** | **[1/2 – 2/3]** | **2/3** | **[2/3 – 1]** | **1** |
| --- | --- | --- | --- | --- | --- | --- | --- | --- | --- |
| **frequency** | 119 | 12 | 22 | 12 | 67 | 12 | 25 | 5 | 116 |

^†^ ‘Sex ratio’ is the proportion of males per plant. Square brackets indicate that range values themselves are not included in the category at issue.

**
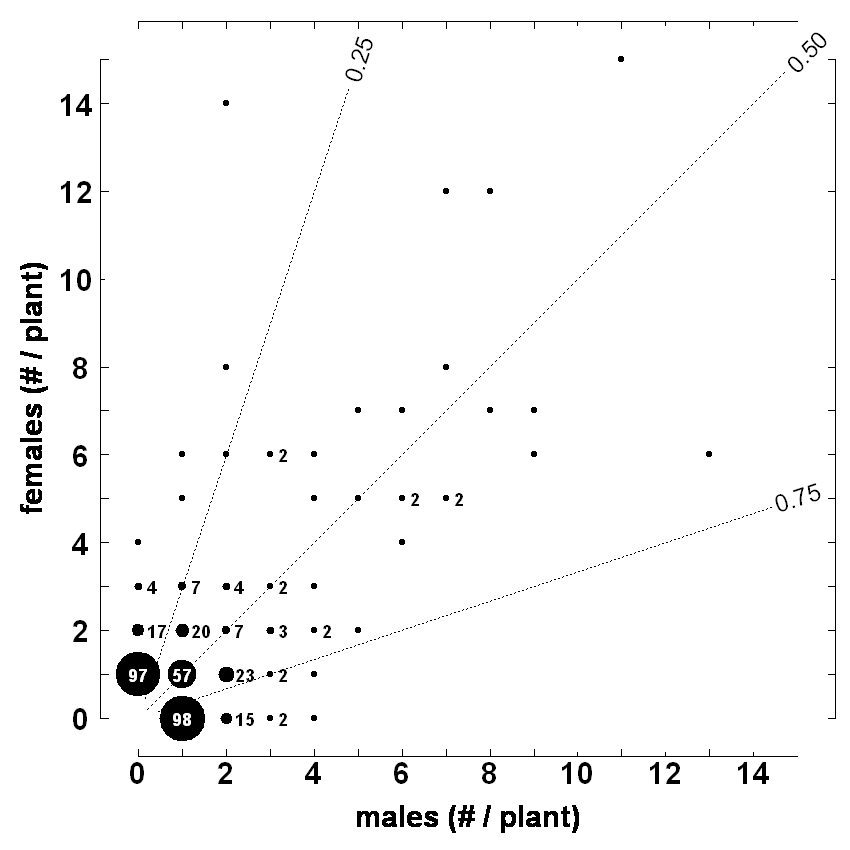
Figure S2.1. Field observations of the number of male and female *Leptinotarsa decemlineata* per plant.** The observations, collected over a period of nine weeks, were made in two insecticide-free potato fields. When combinations (dots) were observed more than once, sample size is both indicated by dot size and printed next to (<20), or in (>20), the dot. Observations of plants without beetles are excluded. Dotted lines indicate some sex ratios to guide the eye. Long tick marks indicate observed values.
