# Supplementary material for: Female Fertilization: Effects of Sex-Specific Density and Sex Ratio Determined Experimentally for Colorado Potato Beetles and Drosophila Fruit Flies
Source: PLoS One. 2013 Apr 12;8(4):e60381. doi: 10.1371/journal.pone.0060381 (PMC3625176; doi:10.1371/journal.pone.0060381)
Supplement: Appendix S3 — Supplementary methods Leptinotarsa decemlineata experiment. (DOCX) [file pone.0060381.s003.docx]

# Appendix S3: Supplementary methods *Leptinotarsa decemlineata* experiment.

**Main paper**: Vahl et al. 2013. **Female fertilization: effects of sex-specific density and sex ratio determined experimentally for Colorado potato beetles and *Drosophila* fruit flies**.

### Subjects and their housing

Between 11 and 18 July 2008 inclusive, we hand-picked about 11 500 4^th^ instar larvae from an insecticide-free potato field of the AAFC Potato Research Center. We transferred these larvae to four mesh-netted field cages (183 x 183 cm and 100 cm high). We covered the floor of these cages with a layer of approximately 15 cm of Pro-Mix soil into which we trans-planted potato plants in high density. This set-up enabled the larvae to complete feeding and initiate pupation in the soil.

From these field cages, we later dug up approximately 4400 pupae, which we brought into the lab. Based on sex-specific characteristics of the seventh abdominal segment [1] and using binoculars (enlargement: 16 to 100 x), we sexed the first 2530 and 1708 pupae as females and males, respectively. We housed these pupae in single-sex groups of up to 25 individuals (mean ± SD: 19.6 ± 1.96) in plastic, cheesecloth-covered cups (473 ml; 11 cm high) containing moist vermiculite, in a chamber with constant air temperature (24°C ± 2°C), humidity (50% ± 10%) and photoperiodic regime (16:8 h light:dark).

We collected adults that emerged from these cups on a daily basis. As mating within the first two days upon emergence does not result in successful fertilization [2], this minimized the chance that adult beetles emerging in these cups got fertilized by erroneously sexed individuals. Using a 10x magnifying glass, we double-checked the sex of emerging adults on the basis of sex-specific characteristics of the last abdominal segment [1], discarding 74 individuals whose sex could not be confirmed. Between 25 July and 7 August inclusive, 1857 females and 1265 males emerged. To ensure their virginity, we kept these individuals in single-sex groups of maximally 24 individuals (19.39 ± 2.46) in plastic containers (2 l; 15 cm high; mesh-lid) provided with fresh potato leaves. These containers were stored in the same chamber and hence under the same climatic conditions as the cups containing pupae. Every second day, we transferred the beetles to clean containers with fresh potato leaves. To minimize the chance that females lost their virginity to erroneously sexed males, we discarded all beetles from containers in which eggs were found, even though virgin *Leptinotarsa decemlineata* are known to occasionally lay eggs [3].

We planted seed potatoes (cultivar ‘Shepody’) in Pro-Mix soil in plant pots of 12.5 cm Ø. We grew these plants in a greenhouse until the day prior to their first use in the experiment; from then onwards, we stored the plants either in the experimental chamber (see below) or in a similar neighbouring chamber. Plants in the experiment were between 21 and 35 days old; they were generally tall (± 50 cm) but narrow (max Ø ± 25 cm), as they were grown adjacently to maximize the number of plants per laboratory surface. We used four potato plants per trial (± 50 cm Ø) to approximate an intermediate sized plant in a commercial potato field.

### Experimental set-up and procedure

We conducted all trials in the same chamber (4.8 x 2.6 m and 2.4 m high) with constant air temperature (24°C ± 2°C), humidity (50% ± 10%) and photoperiodic regime (16:8 h light:dark). We used six pairs of mesh-netted cages to acclimatize beetles to experimental conditions, but only one cage per pair to run the experiment. The cages in each pair were near identical, all ‘experimental’ cages measuring 57 x 57 cm and 60 cm high, with a false, chipboard floor mounted at 12 cm. The centre of this false floor had four slots drilled into it (two on each diagonal and each 4.5 cm from the centre). These slots were sized such that the soil of the completely filled plant pots placed in these slots was exactly at the level of the false floor, with no gap in between. Dust-covered Echotape on the lower 4 cm of the upright sides of all cages obstructed beetles from climbing these sides.

Prior to a daily series of trials, we placed a set of two plants in the centre of all cages, in such a way that their canopy overlapped and did not touch the cage. To create an independent, on-leaf starting-point, we attached a small (5 cm Ø) Petri dish lid with a hairpin to an intermediate-height leaf of each plant. To randomize the assignment of beetles to treatments (see below), we transferred 63 males and 63 females to 126 Petri dishes. Using an *a priori* created randomization scheme, we then brought together same sex beetles that were to participate in the same trial. We placed the Petri dishes containing females and males in pre-selected, paired experimental and acclimation-only cages, respectively. We started the acclimation phase of trials ten minutes after each other, by removing lids from Petri dishes. At the end of the acclimation period, we gathered males and females that were not on a plant, and we placed them in approximately equal numbers on the on-leaf starting-points.

We initiated each trial by carefully transferring the two plants with male beetles to the two (previously covered) unused slots of the experimental cage containing the two plants with female beetles. Long trials started between 11h35 and 12h40; short trials between 15h20 and 16h50.

At the end of each trial we collected the beetles; if we could not find all of them, we kept plants from that trial apart in separate cages, examining them for missing beetles on the day following the trial. On day 5 of the experiment we discovered that most if not all missing beetles had dug themselves in the plant soil, presumably to initiate diapause. Therefore, from day 5 onward, we also searched the soil at the base of the plants. We only reused plants from trials in which we found all beetles, and when defoliation was marginal.

### Treatment levels

We used six levels for both male density and female density, compromising the ability to estimate treatment effects accurately [4, 5] with logistic feasibility. As it is desirable for treatment levels to have a broad range [5, 6], male and female density per plant (1 to 32) encompassed and exceeded the range usually observed both in untreated potato fields and in chemically treated potato fields (highest mean densities: < 2 [7, 8], ≈ 10 [9], and ≈ 15 [10]). Expecting the steepest increase in fertilization probability at low density levels, we distributed the treatment levels according to a geometric series, which places the highest concentration of treatments at low density levels [6].

Based on the results of pilot trials, we chose trial durations such that at least in some trials, some, but not all, females would be fertilized. Mate guarding times can exceed one hour in *Leptinotarsa decemlineata* ([11], Vahl, personal observation), but average copulation and mate guarding time is only about 15 minutes [12, 13]. Therefore, multiple mating bouts could occur even in short trials. As it turned out, the maximum number of females fertilized per male per trial was at least 2 and 6 for short trials and long trials, respectively.

### Statistical analysis

In using logistic regression to analyze the experiment, we assumed effects of all model terms to be additive and linear, and error to be distributed binomially. In analyzing treatment effects on logit-transformed fertilization probability, we assume independence among the binary measurements that make up the binomial estimates of fertilization probability (Collett [14] §6.1). We sail by the risk of correlated binary measurements, because we subjectively judge the data set insufficient to warrant the elaborate, and partly ad hoc, approaches available to model the overdispersion that may result from such a correlation (Collett [14] §6.2 to §6.7).

### Missing values and their treatment

In two trials (short M_4_F_2_ and long M_32_F_4_), treatment levels were not as intended. In both trials, dissection proved one supposed male to be a female. In all analyses, we used the actual rather than the intended treatment levels for these two trials. Expecting the two incorrectly sexed females to have been fertilized from the outset of the experiment, we excluded one fertilized female from the score of these two trials each.

Twenty-nine beetles could not be found at the end of the trials in which they had participated. We later found two of them on the plants of their trial; these two beetles were included in all analyses as if they had not been missing. Nineteen of them (3 males, 12 unfertilized females and 4 fertilized females) were found buried in the soil. The extent to which these beetles had participated in their trial is uncertain, and, therefore, we ran all analyses twice, once including and once excluding them. We did not find the other eight individuals at all (six of them went missing before we started searching the soil for buried beetles on day 5). As four of the missing individuals were females, we could estimate the proportion of females fertilized less accurately in four trials (long M_8_F_8_, long M_32_F_32_, short M_32_F_16_, short M_8_F_32_). Accuracy was similarly reduced for one trial (long M_16_F_32_), for which we lost a spermathecum in the process of dissection.

Excluding buried and missing individuals from the analyses hardly affected the estimated parameter values and their standard deviation. Therefore, we present only the results of the analyses that include the buried and missing individuals.

### Additional analyses

To examine the extent to which parameter estimates depended upon model assumptions, we performed several additional analyses. Comparison with logistic regression models in which the two runs of our experiment were analysed separately, indicated that our decision to analyse the two runs of our experiment together had but negligible effects. Logistic regression models that deviated from the ones presented in the main text only in that the block factors were treated as fixed, gave no indication that estimated parameter effects depended on our assumption to treat the block factors day and cage as random. Likewise, logistic regression models that deviated from the ones presented in the main text only in that all model terms involving either female density or cage were excluded, did not indicate the estimated effects of female density and the block factor cage to be confounded.

Analyses of simulated data showed that with the data structure of the experiment, model deviance could be high even in the absence of overdispersion (conform Collett [14], §6.1). For this reason, and because our sample size was only moderate, we decided against modelling overdispersion.

To evaluate effects of the compound factors ‘total density’ (male density plus female density) and ‘sex ratio’ (the proportion of males) on female fertilization probability, we ran a model identical to that presented in the main text, but with total density and sex ratio, rather than male density and female density, as (continuous, fixed) treatment factors. To improve the spread of treatment levels in this model, we log_2_-transformed the values of total density; we did not transform the values of sex ratio, as treatment levels covered the range of possible sex ratios reasonably well, and no straightforward transformation improved their distribution. Centering the treatment values removed some, but not all, correlation between model terms; the strongest correlation for any combination of centered, fixed model terms was 0.35 (r_Pearson_, absolute value). This implies that full separation of all treatment effects was not possible in this model.

# REFERENCES

1. Pelletier Y (1993) A method for sex determination of the Colorado potato beetle pupa, *Leptinotarsa decemlineata* (Coleoptera: Chrysomelidae). Entomol News 104: 140-142.
2. Alyokhin AV, Ferro DN (1999) Reproduction and dispersal of summer-generation Colorado potato beetle (Coleoptera: Chrysomelidae). Environ Entomol 28: 425-430.
3. Peferoen M, Huybrechts R, Deloof A (1981) Longevity and fecundity in the Colorado potato beetle, *Leptinotarsa decemlineata*. Entomol Exp Appl 29: 321-329.
4. Cox DR (1958) Planning of experiments. New York: John Wiley & Sons, Inc. 308 p.
5. Inouye BD (2001) Response surface experimental designs for investigating interspecific competition. Ecology 82: 2696-2706.
6. Cousens R (1991) Aspects of the design and interpretation of competition (interference) experiments. Weed Technol 5: 664-673.
7. Boiteau G (1986) Effect of planting date and plant spacing on field colonization by Colorado potato beetles, *Leptinotarsa decemlineata* (Say), in New Brunswick. Environ Entomol 15: 311-315.
8. Boiteau G (2005) Within-field spatial structure of Colorado potato beetle (Coleoptera: Chrysomelidae) populations in New Brunswick. Environ Entomol 34: 446-456.
9. Boiteau G (2001) Recruitment by flight and walking in a one-generation Colorado potato beetle (Coleoptera: Chrysomelidae) environment. Environ Entomol 30: 306-317.
10. Noronha C, Cloutier C (1999) Ground and aerial movement of adult Colorado potato beetle (Coleoptera: Chrysomelidae) in a univoltine population. Can Entomol 131: 521-538.
11. Gibson A, Corham RP, Hudson HF, Flock JA (1925) The Colorado potato beetle in Canada. Ottawa: Dominion of Canada, Department of Agriculture. Bulletin 52. 30 p.
12. Boiteau G (1988) Sperm utilization and post-copulatory female-guarding in the Colorado potato beetle, *Leptinotarsa decemlineata*. Entomol Exp Appl 47: 183-187.
13. Radtke MG, Rutowski RL (2002) Variation in the number of sperm transferred during mating among males of the Colorado potato beetle (Coleoptera: Chrysomelidae). J Insect Physiol 48: 1087-1092.
14. Collett D (2003) Modelling binary data. London: Chapman & Hall / CRC. 408 p.
