# Supplementary material for: Female Fertilization: Effects of Sex-Specific Density and Sex Ratio Determined Experimentally for Colorado Potato Beetles and Drosophila Fruit Flies
Source: PLoS One. 2013 Apr 12;8(4):e60381. doi: 10.1371/journal.pone.0060381 (PMC3625176; doi:10.1371/journal.pone.0060381)
Supplement: Appendix S4 — Supplementary results Leptinotarsa decemlineata and Drosophila experiments. (DOCX) [file pone.0060381.s004.docx]

# Appendix S4: Supplementary results *Leptinotarsa decemlineata* and *Drosophila* experiments

**Main paper**: Vahl et al. 2013. **Female fertilization: effects of sex-specific density and sex ratio determined experimentally for Colorado potato beetles and *Drosophila* fruit flies**.

## LEPTINOTARSA DECEMLINEATA

The full data set is presented in Table S4.1.

### Leptinotarsa decemlineata - Effects of male density and female density

Visual inspection of the logistic regression of the ln-transformed odds of fertilization of a female *Leptinotarsa decemlineata* (Fig. S4.1) indicates that, qualitatively, both trial duration and male density had a strong, positive effect on the ln-transformed odds of fertilization, whereas there was no indication of effects of female density or interaction terms. These observations are also supported by test statistics (Table 1). Only trial duration and male density had high estimated parameter values relative to the associated standard error. None of the other explanatory variables had z-values (i.e. Wald statistics; the ratio of estimated effect size over its standard deviation) substantially higher than 1. Effect sizes for trial duration and male density were substantial and unlikely to be due to chance alone. The same signal is given by the confidence intervals associated with the parameter estimates, which included zero for all explanatory variables but trial duration and male density. Likewise, the odds ratios (i.e. the ratios of the odds of fertilization at two treatment levels that differ by exactly one unit) deviated from 1 only for trial duration and male density, indicating that effects of all other explanatory variables on ln-transformed odds of fertilization of a female *Leptinotarsa decemlineata* are likely to have arisen from chance alone.

### Leptinotarsa decemlineata - Effects of total density and sex ratio

Visual inspection of the fertilization results plotted in relation to sex ratio and total density (Fig. S4.2) suggests positive and interacting effects of sex ratio and total density. Logistic regression of effects of total density and sex ratio on the ln-transformed odds of fertilization (Table S4.2) supports this suggestion; females were especially likely to be fertilized when both sex ratio and total density were high. The interacting effect of sex ratio and total density depended itself on trial duration, though only weakly so, as indicated by the rather low parameter estimate for the three-way interaction term, and by its moderate effect on the odds ratio (Table S4.2).

## DROSOPHILA DATA

### Wallace [1] – Effects of sex ratio and total density

Visual inspection of the logistic regression of the ln-transformed odds of fertilization of a female *Drosophila melanogaster sepia* (Fig. S4.3) confirms that, qualitatively, both sex ratio and total density had a strong, positive effect on the ln-transformed odds of fertilization, whereas the interaction effect between these two factors appeared to be weak.

### Wallace [1] – Effects of the total number of males and females

Logistic regression of the ln-transformed odds of fertilization of a female *Drosophila melanogaster sepia* and the densities of males and females in the experiment of Wallace [1] suggests that females were especially likely to be fertilized when many males were around and when few females were around, with a small interaction between these two factors (Table S4.3). Doubling male density led to a 167 % increase of the ln-transformed odds of fertilization of a female, while doubling female density implied a 52 % decrease of these odds. For each of the model terms, though especially for male density and female density, the absolute z-values (i.e. Wald statistics) are considerably higher than 1, indicating that the parameter estimate was substantial relative to its standard error. The 95% confidence intervals for each of these effects do not include zero, lending further support that these estimated effects were not due to chance alone.

Comparing models that varied in whether they included both male density, female density and their interaction term, revealed that parameter estimates for the main effects varied considerably depending on model composition (Table S4.4). The estimated effect size of male density was more than twice as high in models that included both male density and female density as in the model that did not contain female density. The estimated effect size of female density became negative and increased seven-fold upon inclusion of male density. This instability in the estimated effect sizes results from the strong correlation between male and female density in the experiments of Wallace [1]; male and female density are correlated to the same extent in the experiments of Wallace [2].

### Wallace [2] – Effects of sex ratio and the total number of flies

Separately, the twelve logistic fixed effects regression analyses of the experiments presented in Wallace [2] show effects of the total number of flies and sex ratio very similar to those observed in our reanalysis of data from Wallace ([1]; Figs. S4.4, S4.5, S4.6, S4.7, S4.8, S4.9, S4.10, S4.11, S4.12, S4.13, S4.14 and S4.15). Females were invariably more likely to be fertilized when the total number of flies was higher and when the proportion of males was higher. In most experiments with *D. m. sepia* and *D. simulans*, but not in those with *D. m. ebony*, effects of the total number of flies and sex ratio additionally interacted, with stronger effects of the total number of flies at the higher sex ratios and vice versa.

For each of the three study strains, predictive ability of the four-factorial logistic mixed effects regression model relating the ln-transformed odds of fertilization of female *Drosophila* in the experiments of Wallace [2] to the total number of flies and their sex ratio improved upon inclusion of squared terms for both the total number of flies and sex ratio (Table S4.5).

### Wallace [2] – Effects of the number of male and female flies

Four-factorial logistic mixed effects regression of the effects of the number of males and females shows that the ln-transformed odds of fertilization for female in each of the three *Drosophila* strains were higher when more males or fewer females were around (Table S4.6). Jointly, the number of males and females had but a minor effect, which size of which depended on the combination of trial duration and the number of mating chambers in *D. m. sepia*, but not in *D. m. ebony* and *D. simulans* (Table S4.6, Fig. S4.16).

# REFERENCES

1. Wallace B (1985) Mating kinetics in *Drosophila*. Behav Sci 30: 149-154.
2. Wallace B (1990) Male male interactions and mating kinetics in *Drosophila*. Behav Genet 20: 405-421.
3. Collett D (2003) Modelling binary data. London: Chapman & Hall / CRC. 408 p.

# SUPPLEMENTARY INFORMATION

Appendix S3: Supplementary methods *Leptinotarsa decemlineata* experiment.

# TABLES

**Table S4.1.** *Leptinotarsa decemlineata* data^†^.

| **TD** | **M** | **F** | **d** | **c** | **π** |  | **TD** | **M** | **F** | **d** | **c** | **π** |  | **TD** | **M** | **F** | **d** | **c** | **π** |
| --- | --- | --- | --- | --- | --- | --- | --- | --- | --- | --- | --- | --- | --- | --- | --- | --- | --- | --- | --- |
| 22 | 32 | 16 | 1 | 1 | 0.88 |  | 22 | 8 | 2 | 5 | 1 | 0.50 |  | 22 | 2 | 2 | 9 | 1 | 0.50 |
| 22 | 16 | 2 | 1 | 2 | 1.00 |  | 22 | 31 | 5 | 5 | 2 | 1.00 |  | 22 | 1 | 4 | 9 | 2 | 0.75 |
| 22 | 8 | 4 | 1 | 3 | 0.25 |  | 22 | 16 | 16 | 5 | 3 | 0.94 |  | 22 | 32 | 8 | 9 | 3 | 1.00 |
| 22 | 4 | 8 | 1 | 4 | 1.00 |  | 22 | 1 | 1 | 5 | 4 | 0.00 |  | 22 | 16 | 32 | 9 | 4 | 0.74 |
| 22 | 2 | 1 | 1 | 5 | 1.00 |  | 22 | 4 | 32 | 5 | 5 | 0.53 |  | 22 | 8 | 1 | 9 | 5 | 1.00 |
| 22 | 1 | 32 | 1 | 6 | 0.03 |  | 22 | 2 | 8 | 5 | 6 | 0.50 |  | 22 | 4 | 16 | 9 | 6 | 0.88 |
| 3 | 16 | 16 | 2 | 1 | 0.00 |  | 3 | 4 | 32 | 6 | 1 | 0.03 |  | 3 | 1 | 32 | 10 | 1 | 0.06 |
| 3 | 4 | 4 | 2 | 2 | 0.00 |  | 3 | 8 | 1 | 6 | 2 | 1.00 |  | 3 | 2 | 8 | 10 | 2 | 0.00 |
| 3 | 2 | 1 | 2 | 3 | 0.00 |  | 3 | 1 | 8 | 6 | 3 | 0.00 |  | 3 | 4 | 16 | 10 | 3 | 0.13 |
| 3 | 32 | 32 | 2 | 4 | 0.22 |  | 3 | 2 | 16 | 6 | 4 | 0.06 |  | 3 | 8 | 2 | 10 | 4 | 0.00 |
| 3 | 1 | 2 | 2 | 5 | 0.00 |  | 3 | 32 | 4 | 6 | 5 | 0.00 |  | 3 | 16 | 4 | 10 | 5 | 0.50 |
| 3 | 8 | 8 | 2 | 6 | 0.00 |  | 3 | 16 | 2 | 6 | 6 | 0.00 |  | 3 | 32 | 1 | 10 | 6 | 0.00 |
| 22 | 16 | 4 | 3 | 1 | 0.75 |  | 22 | 4 | 2 | 7 | 1 | 0.00 |  | 22 | 1 | 2 | 11 | 1 | 0.00 |
| 22 | 4 | 1 | 3 | 2 | 0.00 |  | 22 | 8 | 32 | 7 | 2 | 0.63 |  | 22 | 2 | 16 | 11 | 2 | 0.25 |
| 22 | 2 | 32 | 3 | 3 | 0.34 |  | 22 | 1 | 16 | 7 | 3 | 0.13 |  | 22 | 4 | 4 | 11 | 3 | 0.00 |
| 22 | 32 | 2 | 3 | 4 | 1.00 |  | 22 | 2 | 4 | 7 | 4 | 0.25 |  | 22 | 8 | 8 | 11 | 4 | 0.86 |
| 22 | 1 | 8 | 3 | 5 | 0.00 |  | 22 | 32 | 1 | 7 | 5 | 1.00 |  | 22 | 16 | 1 | 11 | 5 | 1.00 |
| 22 | 8 | 16 | 3 | 6 | 0.63 |  | 22 | 16 | 8 | 7 | 6 | 0.63 |  | 22 | 32 | 32 | 11 | 6 | 0.91 |
| 3 | 8 | 32 | 4 | 1 | 0.19 |  | 3 | 2 | 32 | 8 | 1 | 0.00 |  | 3 | 32 | 8 | 12 | 1 | 0.50 |
| 3 | 32 | 16 | 4 | 2 | 0.40 |  | 3 | 1 | 16 | 8 | 2 | 0.00 |  | 3 | 16 | 32 | 12 | 2 | 0.25 |
| 3 | 16 | 8 | 4 | 3 | 0.50 |  | 3 | 32 | 2 | 8 | 3 | 1.00 |  | 3 | 8 | 16 | 12 | 3 | 0.31 |
| 3 | 1 | 4 | 4 | 4 | 0.00 |  | 3 | 16 | 1 | 8 | 4 | 0.00 |  | 3 | 3 | 3 | 12 | 4 | 0.00 |
| 3 | 4 | 1 | 4 | 5 | 0.00 |  | 3 | 8 | 4 | 8 | 5 | 0.00 |  | 3 | 2 | 4 | 12 | 5 | 0.25 |
| 3 | 2 | 2 | 4 | 6 | 0.00 |  | 3 | 4 | 8 | 8 | 6 | 0.13 |  | 3 | 1 | 1 | 12 | 6 | 0.00 |

† Columns indicate trial duration ‘TD’, male density ‘M’, female density ‘F’, day ‘d’, and cage ‘c’, and the percentage of females fertilized ‘π’ at the end of each trial (rows). Values include buried and missing beetles (see Appendix S3).

**Table S4.2.** Results of the (logistic) regression model of the effects of total density and sex ratio on the ln-transformed odds of fertilization for female *Leptinotarsa decemlineata*^†^.

| n = 72, AIC = 146.14 **Treatment effects (fixed)** | **β ± s.e.** | **95% CI (low, high)** | **odds ratio** | **z- value** |
| --- | --- | --- | --- | --- |
| **whole plot** |  |  |  |  |
| constant | **-0.71 ± 0.23** | (-1.16, -0.26) | - | - |
| trial duration ‘TD’ | **0.14 ± 0.02** | ( 0.10, 0.19) | 1.15 | 5.94 |
| **subplot** |  |  |  |  |
| log_2_(total density) ‘T’ | **0.70 ± 0.14** | ( 0.43, 0.98) | 2.02 | 5.06 |
| sex ratio ‘S’ | **3.01 ± 0.69** | (1.65, 4.36) | 20.20 | 4.36 |
| TD · T | 0.02 ± 0.01 | (-0.01, 0.05) | 1.02 | 1.39 |
| TD · S | 0.02 ± 0.07 | (-0.12, 0.16) | 1.02 | 0.28 |
| T · S | **0.99 ± 0.61** | (-0.21, 2.18) | 2.69 | 1.62 |
| TD · T · S | 0.10 ± 0.06 | (-0.02, 0.23) | 1.11 | 1.59 |

^†^ Total density is the sum of male density and female density; sex ratio is the proportion of males. Parameter estimates ‘β’, their standard error ‘s.e.’, and variances ‘σ^2^’ and their standard deviation ‘s.d.’ were computed using the ‘lmer’ function in R. Confidence intervals ‘CI’ of parameter estimates were computed as β ± z_α,2_ · se(β), the ‘odds ratio’, that is, the ratio of the odds of fertilization at two treatment values that differ exactly one unit, was computed as exp(β), and the ‘z-values’ were computed as β / se(β), all conform Collett [3]. Effects that are substantial relative to their standard error are presented in bold to guide the eye. Random effects (σ^2^) of the block factors experimental day (nested within TD) and cage were 0.24 and 0.00, respectively.**Table S4.3.** Results of the (logistic) regression model of the ln-transformed odds of fertilization for female *Drosophila* *melanogaster sepia* strains in relation to the density of males and females in the experiment of Wallace [1]^†^.

| n ≥ 103, AIC = 138.09 **Treatment effects (fixed)** | **β ± s.e.** | **95% CI (low, high)** | **odds ratio** | **z-value** |
| --- | --- | --- | --- | --- |
| constant | -0.09 ± 0.06 | (-0.038, 0.208) | - | - |
| log_2_(male density) ‘M’ | **0.98 ± 0.05** | ( 0.891, 1.077) | 2.67 | 20.68 |
| log_2_(female density) ‘F’ | -**0.73 ± 0.05** | (-0.825, -0.636) | 0.48 | -15.10 |
| M · F | -**0.05 ± 0.02** | (-0.088, -0.018) | 0.95 | -2.96 |

^†^ Details of the computation and interpretation of the presented statistics can be found in the footnote to Table S4.2. Effects that are substantial relative to their standard error are presented in bold to guide the eye.

**Table S4.4.** Parameter estimates of various logistic regression models of the ln-transformed odds of fertilization for female *Drosophila melanogaster sepia* in relationship to the density of males and females in the experiment of Wallace [1]^†^.

|  | **model composition** | | | |
| --- | --- | --- | --- | --- |
|  | **c + M** | **c + F** | **c + M + F** | **c + M + F + M · F** |
| constant ‘c’ | -0.40 ± 0.04 | -0.60 ± 0.05 | -0.04 ± 0.05 | 0.09 ± 0.06 |
| log_2_(male density) ‘M’ | 0.42 ± 0.03 | 0.42 - 0.03 | 0.95 ± 0.05 | 0.98 ± 0.05 |
| log_2_(female density) ‘F’ | 0.42 - 0.03 | 0.10 ±0.03 | -0.69 ± 0.05 | -0.73 ± 0.05 |
| M · F | 0.42 - 0.03 | 0.42 - 0.03 | 0.42 - 0.03 | -0.05 ± 0.02 |

^†^ Presented values indicate parameter estimates and their standard error for the treatment factors (rows) in various models (columns) that varied in whether they accounted for effects of the density of males and females and their interaction.

**Table S4.5.** Comparison of the performance (AIC-values) of various second-order mixed effects logistic regression models of the ln-transformed odds of fertilization of female *Drosophila* in the experiments of Wallace [2]^†^.

| **Treatment effects (fixed)** | **Block effect (random)** | ***D. m. ebony*** | ***D. m. sepia*** | ***D. simulans*** |
| --- | --- | --- | --- | --- |
| nC · TD · N · S | 1 \| Exp_ID | 384.10 | 312.90 | 172.48 |
| nC · TD · N · S + N^2^ | 1 \| Exp_ID | 285.02 | 275.93 | 156.31 |
| nC · TD · N · S + S^2^ | 1 \| Exp_ID | 349.51 | 263.66 | 156.92 |
| nC · TD · N · S + N^2^ + S^2^ | 1 \| Exp_ID | 242.39 | 212.82 | 137.48 |

^†^ For each of the three study strains the AIC-values are presented for extensions of the four-factorial mixed effects models containing the number of mating chambers ‘nC’, trial duration ‘TD’, the total number of flies ‘N’, and sex ratio ‘S’, and the random intercept factor experimental identity code ‘1 | Exp_ID’. The various models differ in whether additionally a squared term is included for the total number of flies, sex ratio, both these terms, or neither of them.

**Table S4.6.** Results of the (logistic) regression model of the ln-transformed odds of fertilization for females of three *Drosophila* strains in relation to the total number of males and females in the experiments of Wallace [2]^†^.

|  | ***D. m. ebony*** (n ≥ 1164) | ***D. m. sepia*** (n ≥ 1117) | ***D. simulans*** (n ≥ 1006) |
| --- | --- | --- | --- |
| **Treatment effects (fixed)** |  |  |  |
| **whole plot** |  |  |  |
| constant | **-0.86 ± 0.02** | **0.15 ± 0.02** | **-0.79 ± 0.02** |
| number of chambers ‘nC’ | **-0.13 ± 0.04** | **-0.41 ± 0.04** | **-0.35 ± 0.05** |
| trial duration ‘TD’ | **1.38 ± 0.09** | **2.19 ± 0.08** | **1.61 ± 0.10** |
| nC · TD | **0.59 ± 0.18** | **-1.17 ± 0.17** | **-1.34 ± 0.19** |
| **subplot** |  |  |  |
| log_2_(total number of males) ‘nM’ | **0.73 ± 0.02** | **0.84 ± 0.01** | **0.70 ± 0.02** |
| log_2_(total number of females) ‘nF’ | **-0.34 ± 0.02** | **-0.59 ± 0.01** | **-0.35 ± 0.02** |
| nM · nF | **-0.06 ± 0.01** | -0.03 ± 0.01 | -0.02 ± 0.01 |
| nC · nM | **-0.05 ± 0.03** | **-0.10 ± 0.03** | **-0.10 ± 0.03** |
| nC · nF | **0.09 ± 0.03** | **0.14 ± 0.03** | **0.12 ± 0.03** |
| TD · nM | **0.19 ± 0.07** | **0.22 ± 0.06** | 0.03 ± 0.07 |
| TD · nF | 0.02 ± 0.07 | **-0.28 ± 0.06** | -0.03 ± 0.07 |
| nC · nM · nF | 0.02 ± 0.01 | 0.01 ± 0.01 | 0.00 ± 0.01 |
| TD · nM · nF | -0.02 ± 0.03 | **-0.07 ± 0.02** | 0.02 ± 0.03 |
| nC · TD · nM | **0.33 ± 0.13** | **-0.36 ± 0.11** | **-0.23 ± 0.14** |
| nC · TD · nF | **-0.25 ± 0.13** | **0.37 ± 0.12** | **0.35 ± 0.14** |
| nC · TD · nM · nF | 0.01 ± 0.05 | **0.22 ± 0.04** | 0.01 ± 0.05 |
| **Block effect (random)** |  |  |  |
| experiment ‘Exp_ID’ | 0.00 | 0.00 | 0.00 |

^†^ Presented values indicate parameter estimates and their standard error for the treatment factors, and variance for the block factor. Substantial effects with a relatively low standard error are presented in bold to guide the eye. Note that, to ease interpretation, the unit of trial duration in these regression models only was hour rather than minute.**
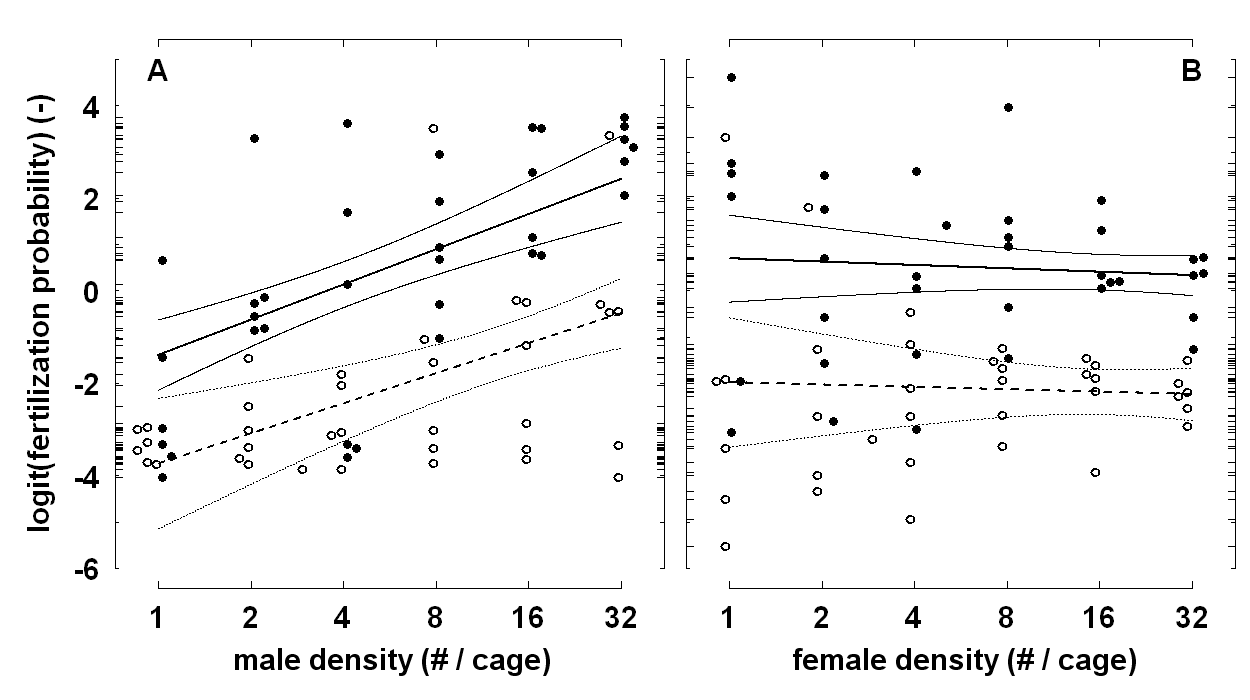
Figure S4.1. The ln-transformed odds (i.e. logits) of fertilization of a female *Leptinotarsa decemlineata.*** The odds of fertilization are related to male density (panel A) and female density (panel B). Open and filled symbols depict single observations from short trials and long trials, respectively, after variation accounted for by model terms other than the ones depicted in the panel at issue has been taken out. Superimposed are the fitted linear regression lines based only on the model terms depicted in the panel at issue (thick lines), with dotted lines and solid lines presenting predictions for short trials and long trials, respectively. The approximate 95 % confidence intervals (Collett [3] §3.15) of the fitted models are indicated by thin lines. Long tick marks indicate treatment levels and response values. Bracketed information in the axis labels concerns the dimension of the variable at issue (´-´ indicating dimensionless). For representational purposes, results of short trials and long trials, as well as results overlapping within trial duration, have been slightly shifted horizontally. Note the log-scale of the x-axis.

**
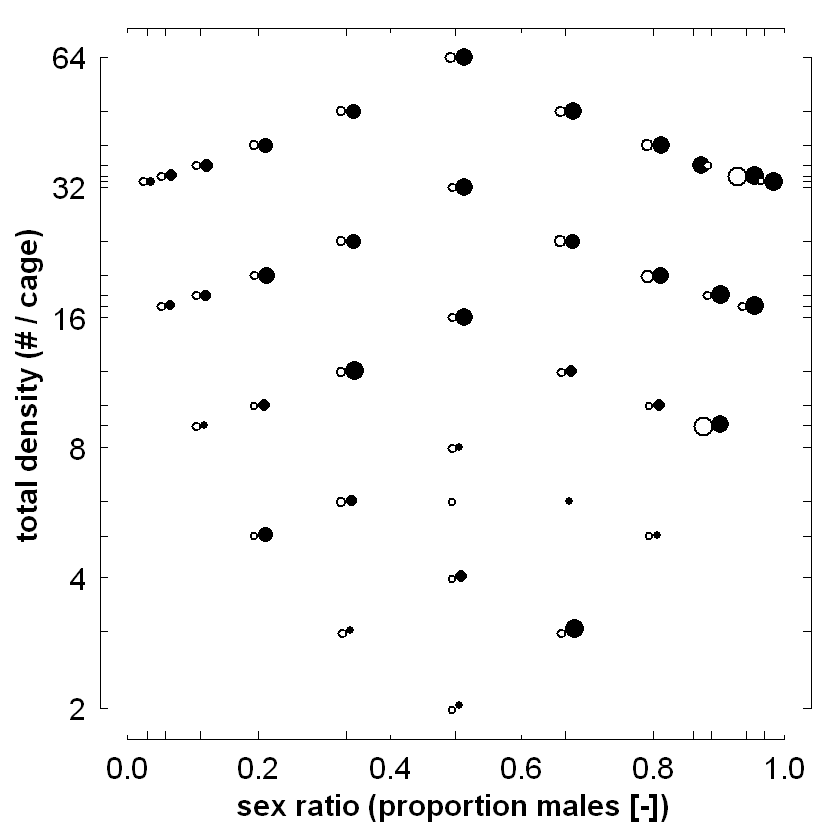
**

**Figure S4.2**. **Proportion of female *Leptinotarsa decemlineata* fertilized at different combinations of sex ratio and total density.** Open and filled symbols depict single observations from short (3h) trials and long (22h) trials, respectively. Symbol size indicates the proportion of females fertilized, with the smallest and largest symbols corresponding to none and all of the females being fertilized, respectively. Long Tick marks indicate treatment levels. For representational purposes, results of short trials and long trials have been slightly shifted diagonally. Note that in two trials (short S_0.67_,T_6_ and long S_0.89_,T_36_), treatment levels were not as intended; dissection proved one supposed male to be a female (see Appendix S3).

**
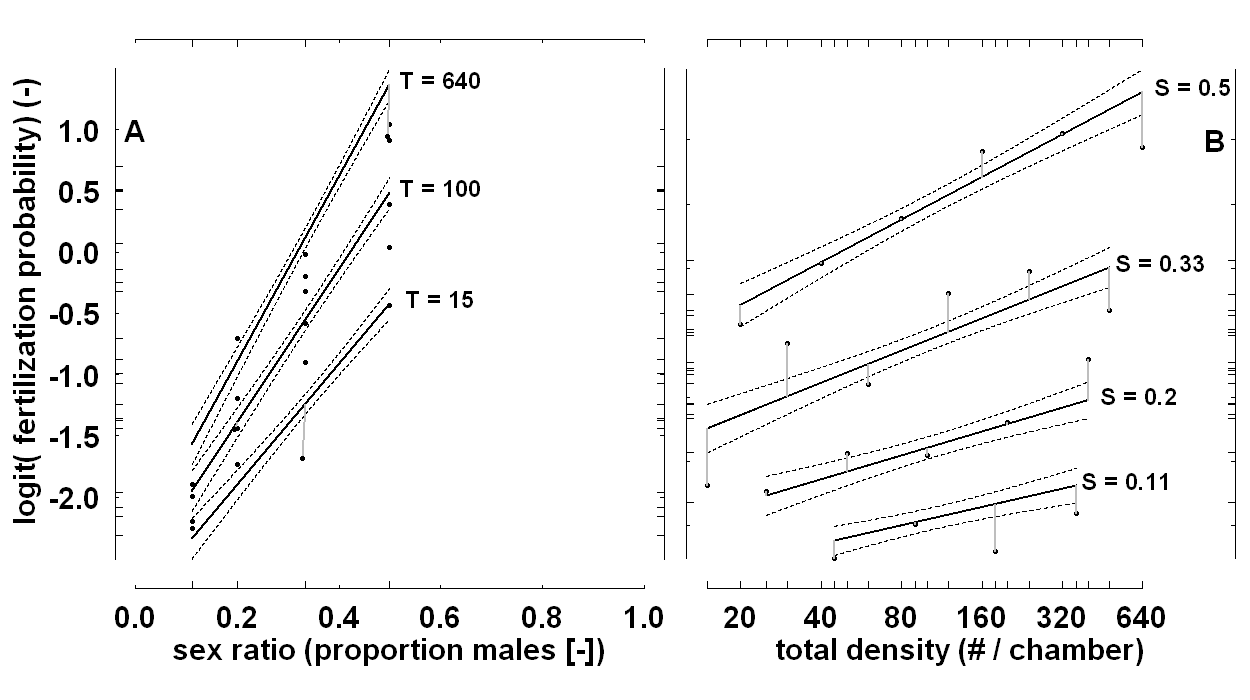
Figure S4.3. The ln-transformed odds (i.e. logits) of fertilization of a female *Drosophila* (data from Wallace [1]).** The odds of fertilization are related to sex ratio (panel A) and total density (panel B). Superimposed are the fitted linear regression lines (continuous lines) and approximate 95 % confidence intervals (dotted lines; Collett [3] §3.15) calculated at specific levels of total density ‘T’ or sex ratio ‘S’. Grey lines show residuals associated with the depicted regression lines (for representational purposes, corresponding observations have been slightly shifted horizontally in panel A). Long tick marks indicate treatment levels and response values. Note the log-scale of the x-axis in panel B.

**
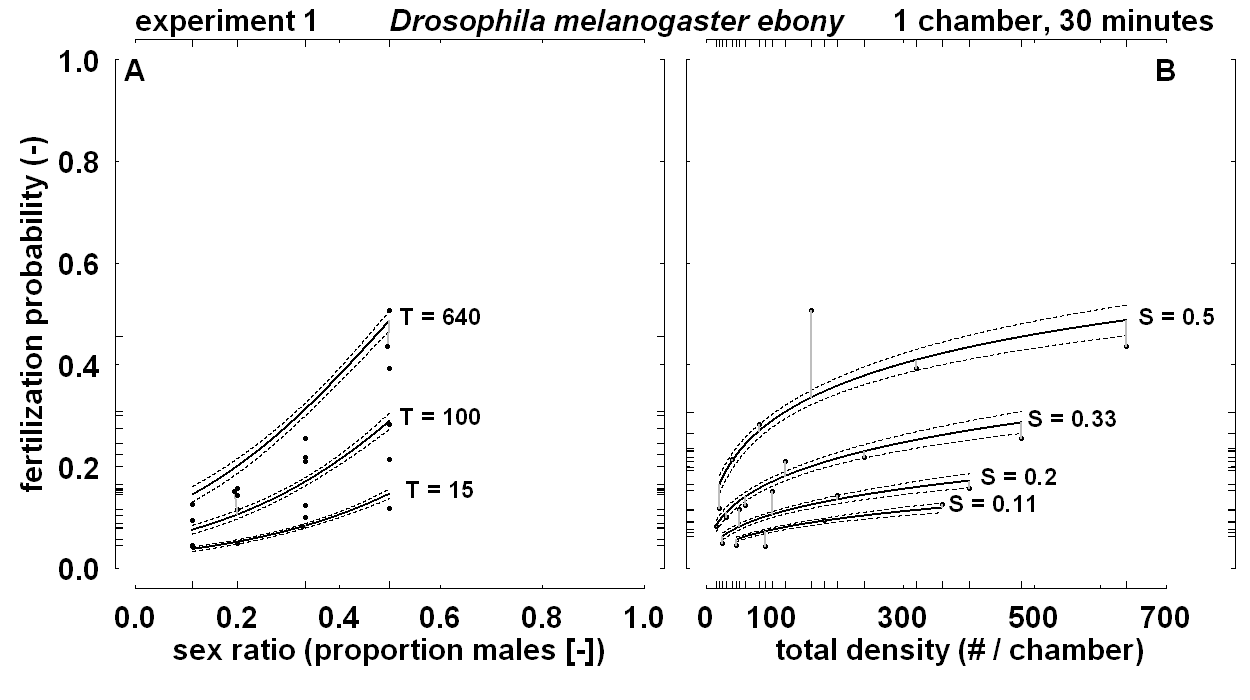
**

**Figure S4.4. Fertilization probability of female *Drosophila* *melanogaster ebony* (30 minutes, one chamber).** Data is from Wallace [2] and concerns the experiment in which flies were together in one experimental chamber for 30 minutes. Presented are back-transformed values in relation to sex ratio (panel A) and total density (panel B). Superimposed are the back-transformed fitted linear regression lines (continuous lines) and 95 % confidence intervals (dotted lines) calculated at specific levels of total density ‘T’ or sex ratio ‘S’. Grey lines show residuals associated with the depicted regression lines (for representational purposes, corresponding observations have been slightly shifted horizontally in panel A). Long tick marks indicate treatment levels and response values. Note the log-scale of the x-axis in panel B.

**
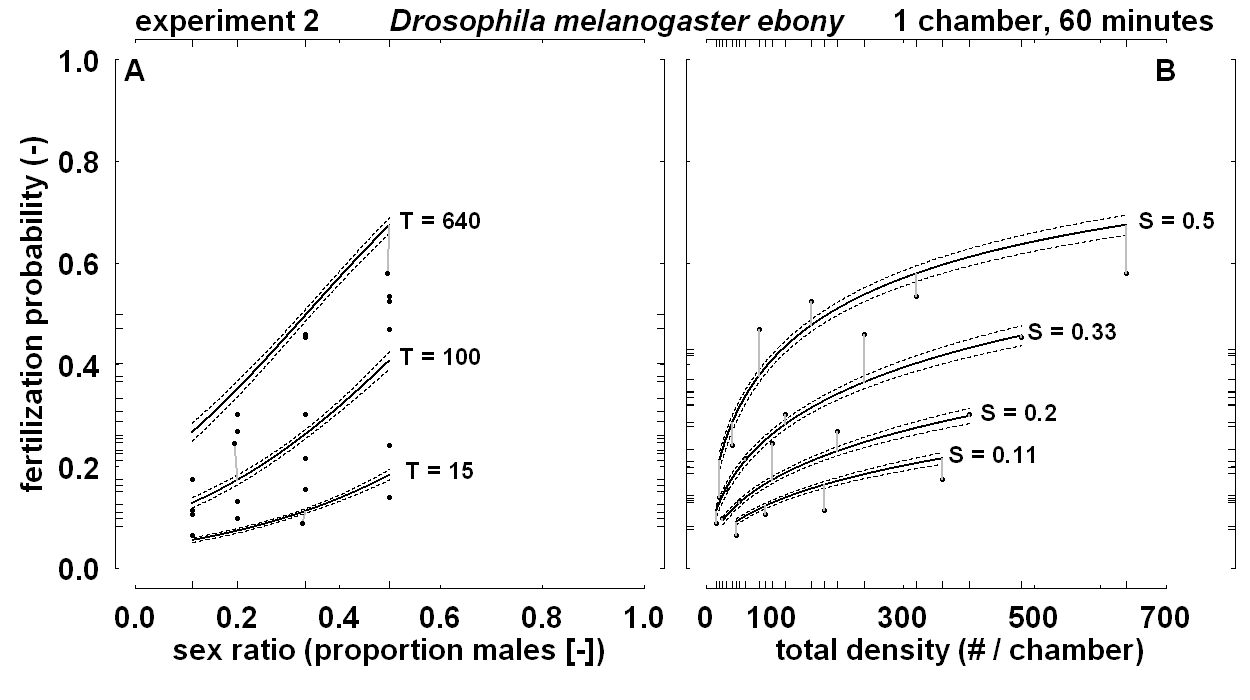
**

**Figure S4.5. Fertilization probability of female *Drosophila* *melanogaster ebony* (60 minutes, one chamber).** Data is from Wallace [2] and concerns the experiment in which flies were together in one experimental chamber for 60 minutes. Composition as in Figure S4.4.

**
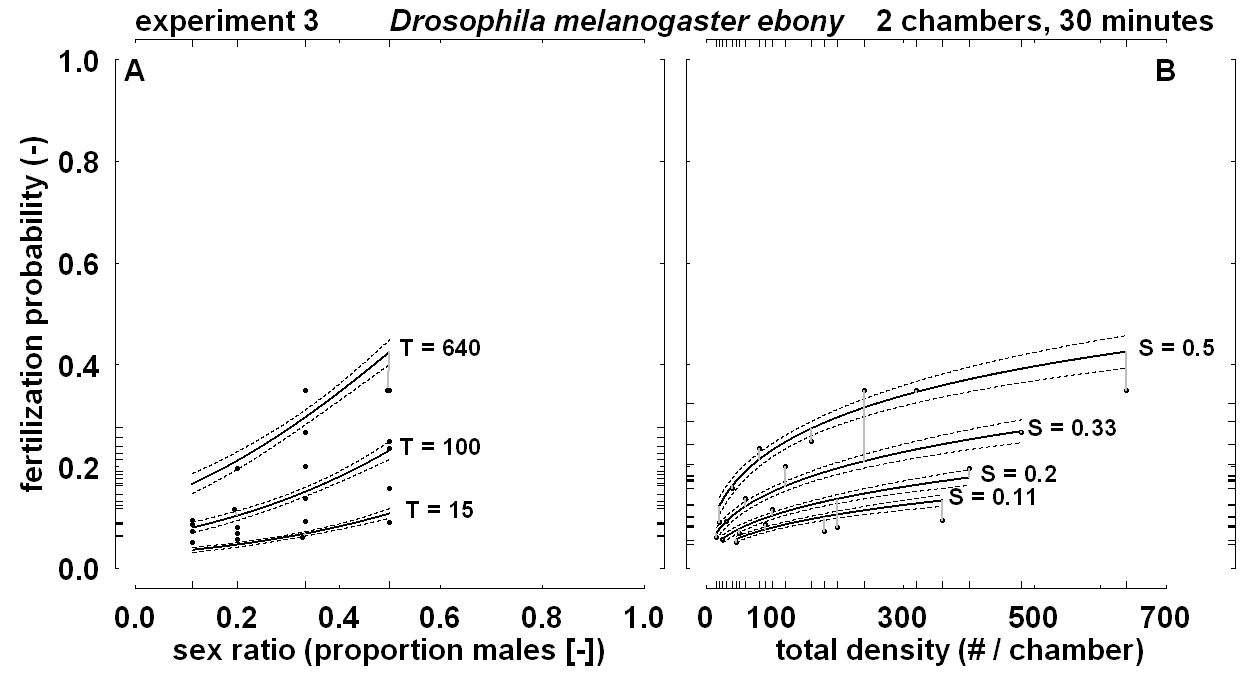
**

**Figure S4.6. Fertilization probability of female *Drosophila* *melanogaster ebony* (30 minutes, two chambers).** Data is from Wallace [2] and concerns the experiment in which flies were together in two experimental chambers for 30 minutes. Composition as in Figure S4.4.

**
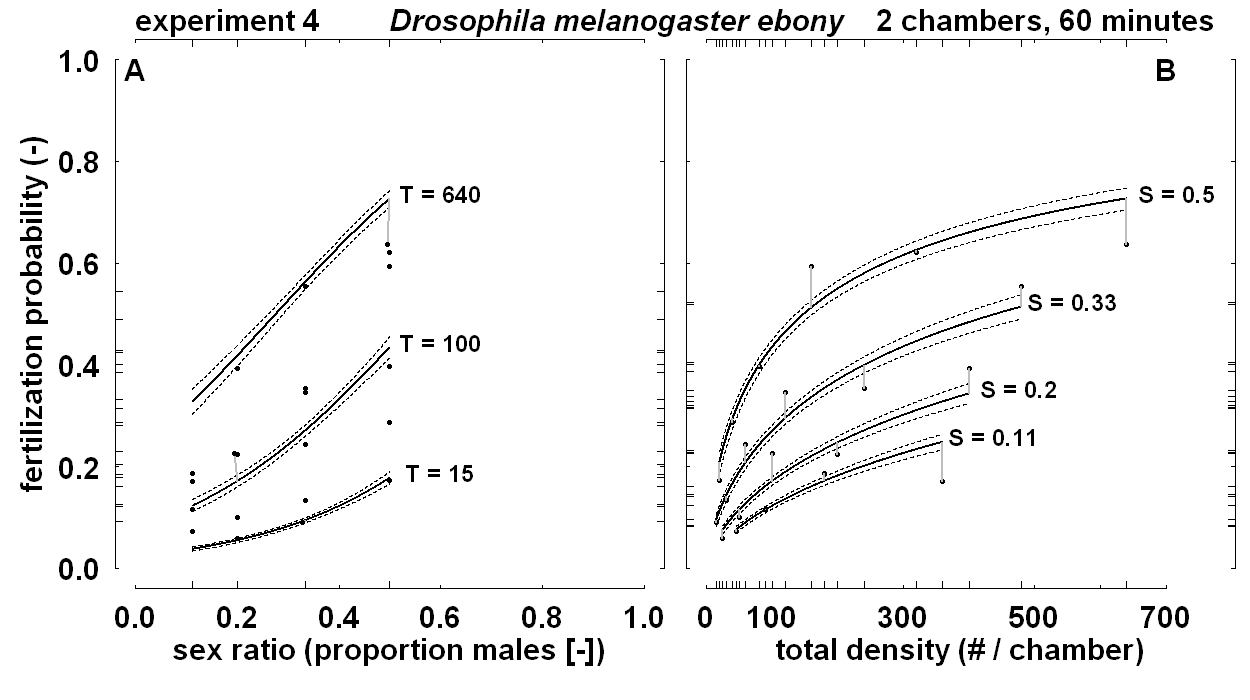
**

**Figure S4.7. Fertilization probability of female *Drosophila* *melanogaster ebony* (60 minutes, two chambers).** Data is from Wallace [2] and concerns the experiment in which flies were together in two experimental chambers for 60 minutes. Composition as in Figure S4.4.

**
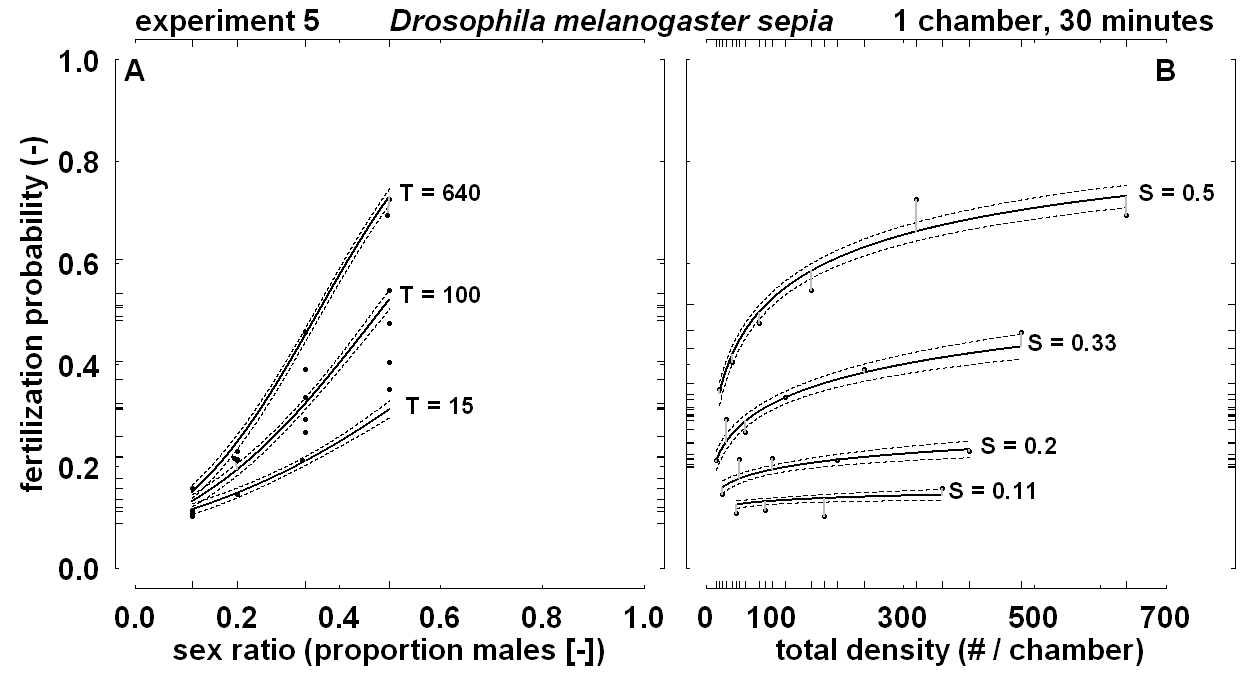
**

**Figure S4.8. Fertilization probability of female *Drosophila* *melanogaster sepia* (30 minutes, one chamber).** Data is from Wallace [2] and concerns the experiment in which flies were together in one experimental chamber for 30 minutes. Composition as in Figure S4.4.

**
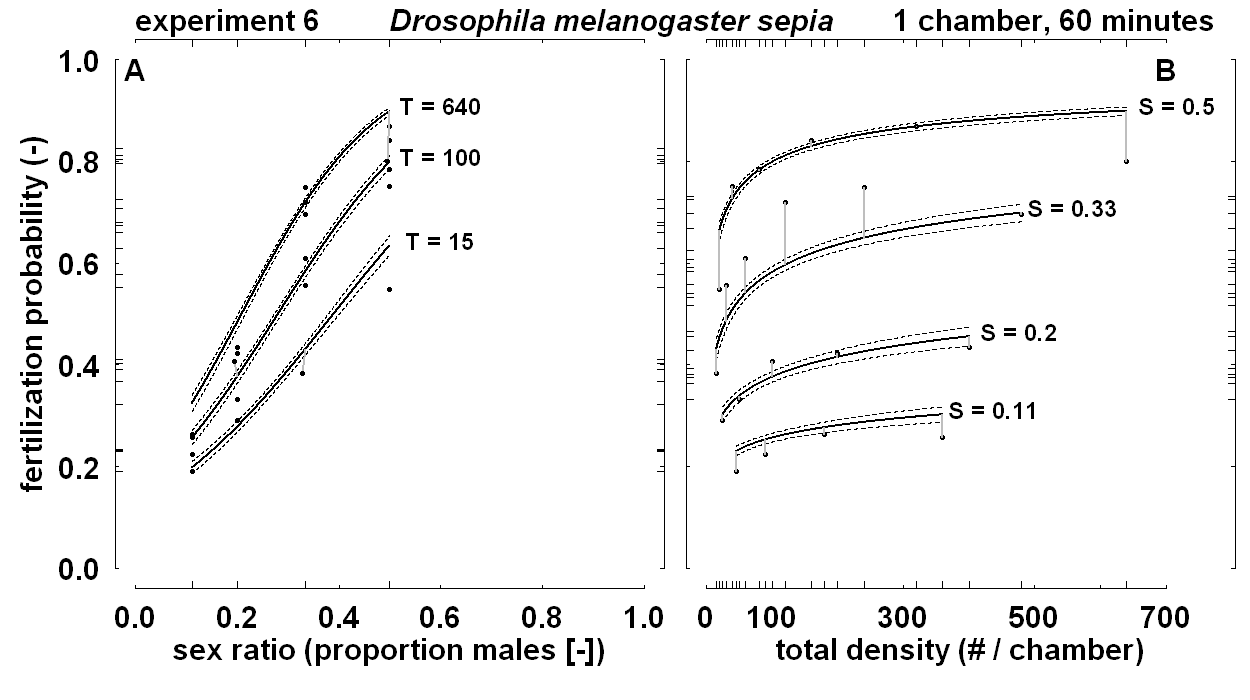
**

**Figure S4.9. Fertilization probability of female *Drosophila* *melanogaster sepia* (60 minutes, one chamber).** Data is from Wallace [2] and concerns the experiment in which flies were together in one experimental chamber for 60 minutes. Composition as in Figure S4.4.

**
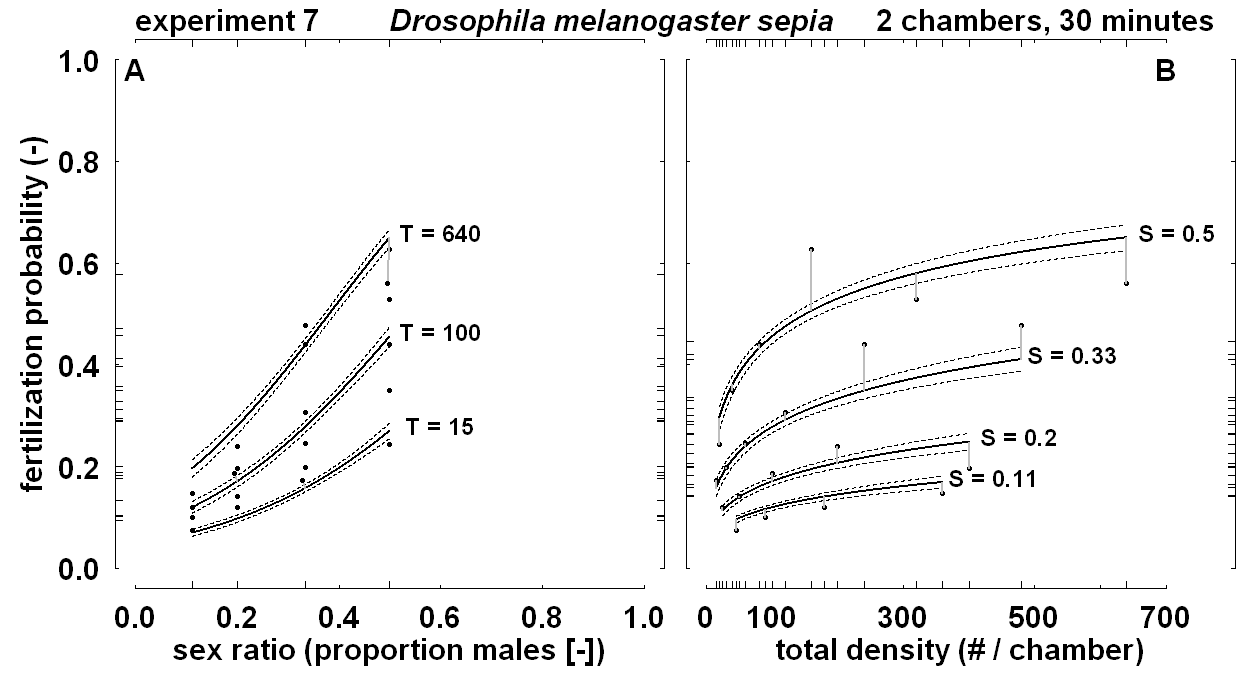
**

**Figure S4.10. Fertilization probability of female *Drosophila* *melanogaster sepia* (30 minutes, two chambers).** Data is from Wallace [2] and concerns the experiment in which flies were together in two experimental chambers for 30 minutes. Composition as in Figure S4.4.

**
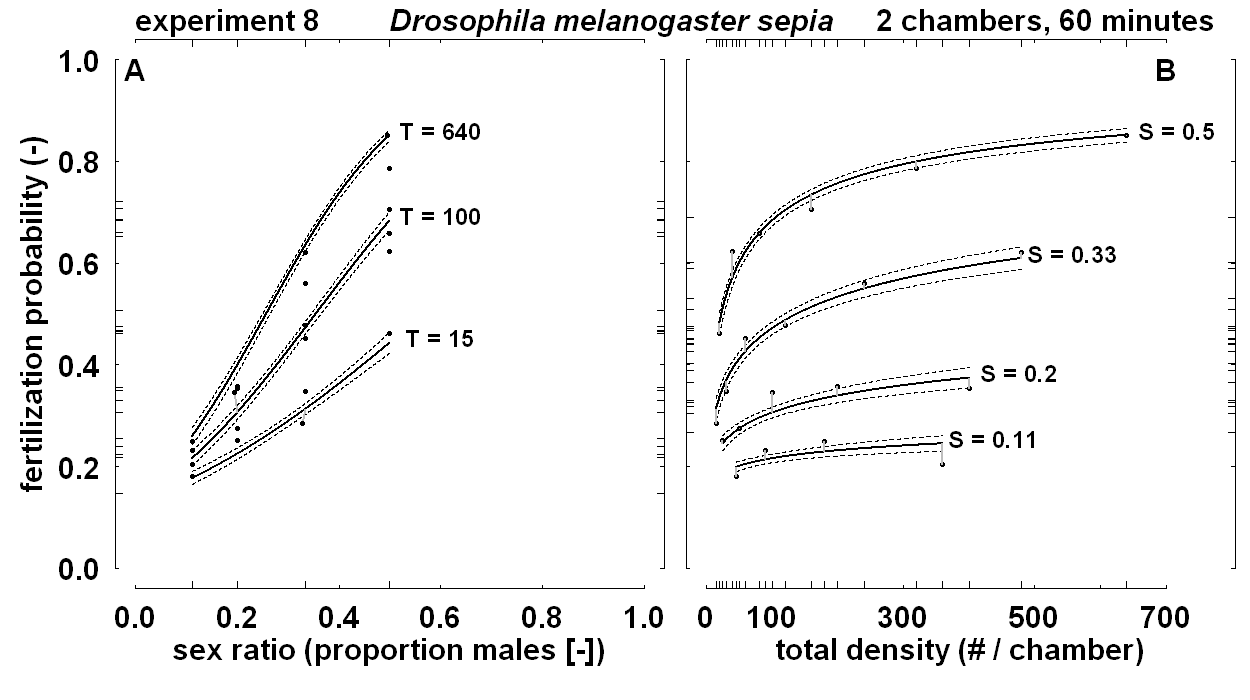
**

**Figure S4.11. Fertilization probability of female *Drosophila* *melanogaster sepia* (60 minutes, two chambers).** Data is from Wallace [2] and concerns the experiment in which flies were together in two experimental chambers for 60 minutes. Composition as in Figure S4.4.

**
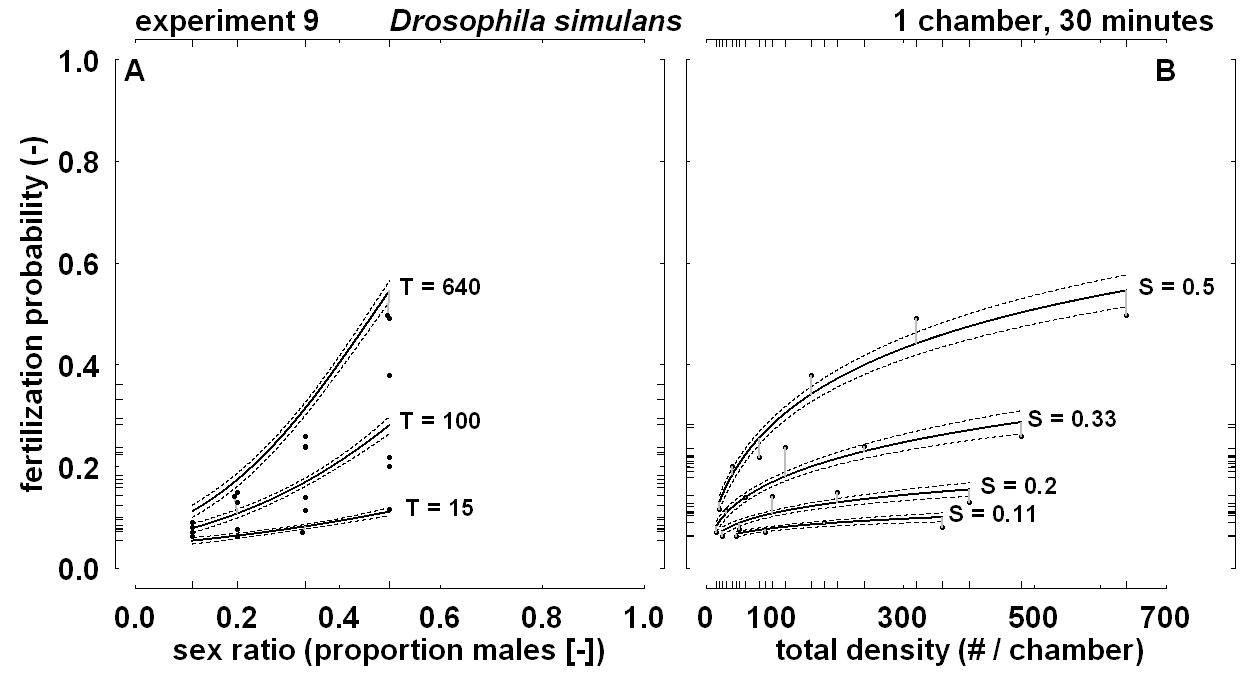
**

**Figure S4.12. Fertilization probability of female *Drosophila* *simulans* (30 minutes, one chamber).** Data is from Wallace [2] and concerns the experiment in which flies were together in one experimental chamber for 30 minutes. Composition as in Figure S4.4.

**
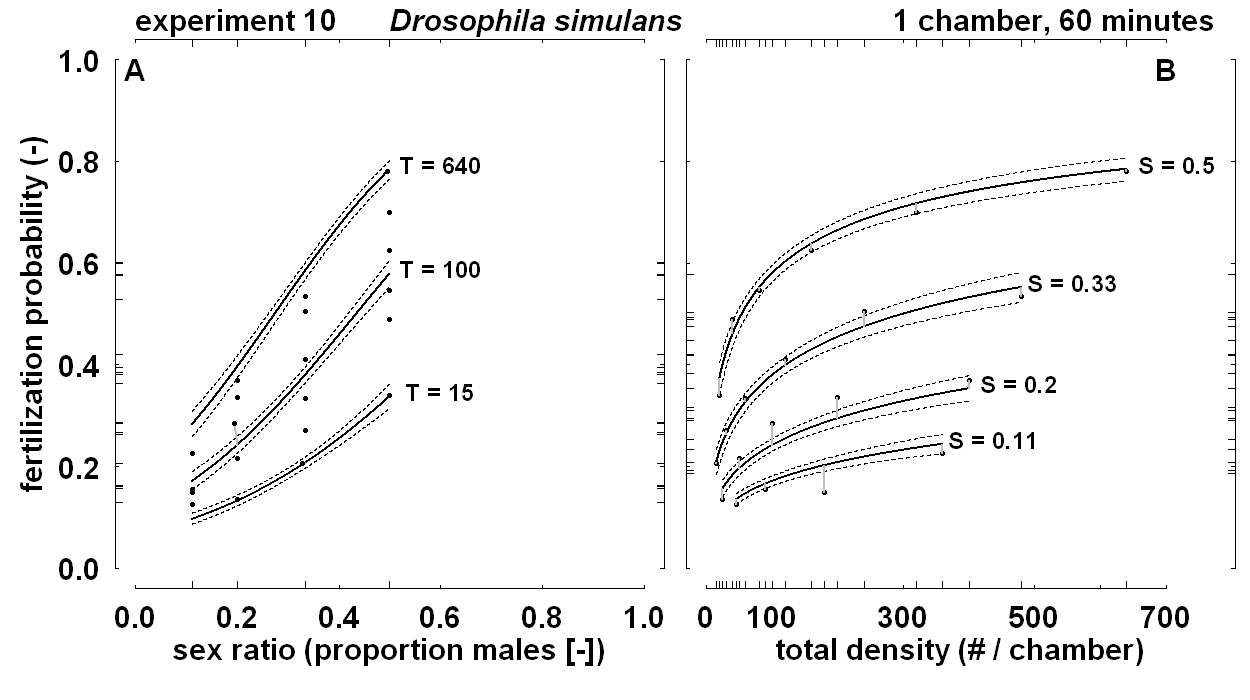
**

**Figure S4.13. Fertilization probability of female *Drosophila* *simulans* (60 minutes, one chamber).** Data is from Wallace [2] and concerns the experiment in which flies were together in one experimental chamber for 60 minutes. Composition as in Figure S4.4.

**
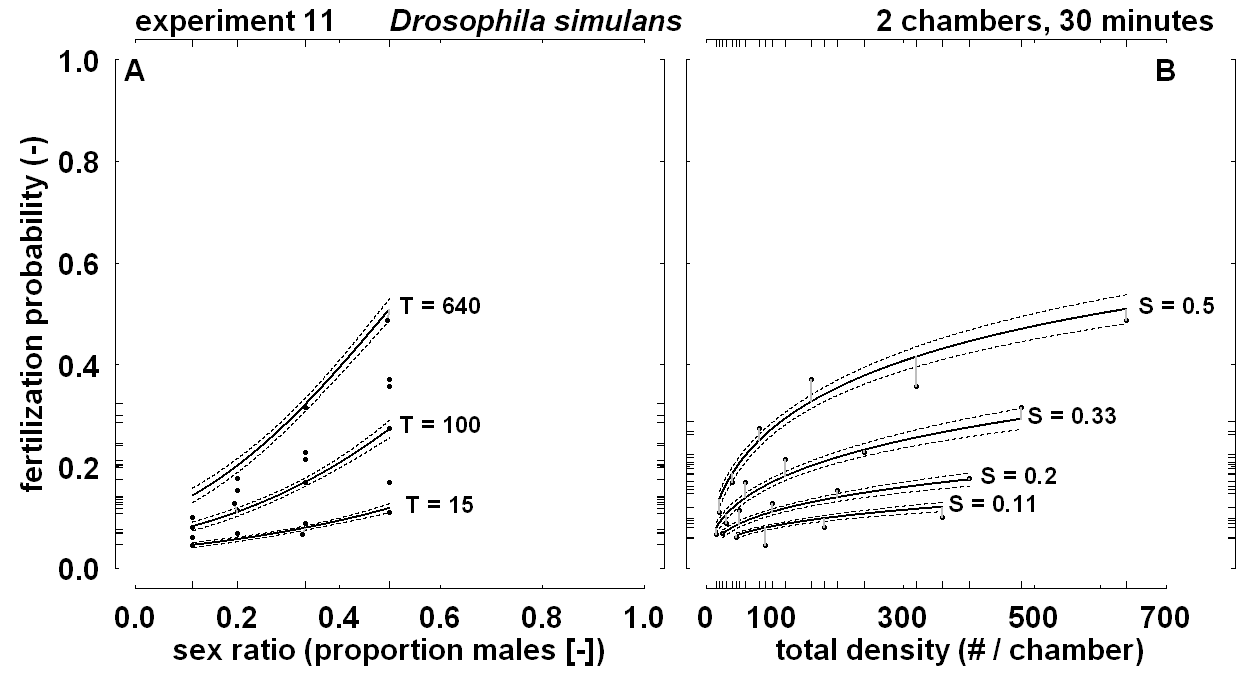
**

**Figure S4.14. Fertilization probability of female *Drosophila* *simulans* (30 minutes, two chambers).** Data is from Wallace [2] and concerns the experiment in which flies were together in two experimental chambers for 30 minutes. Composition as in Figure S4.4.

**
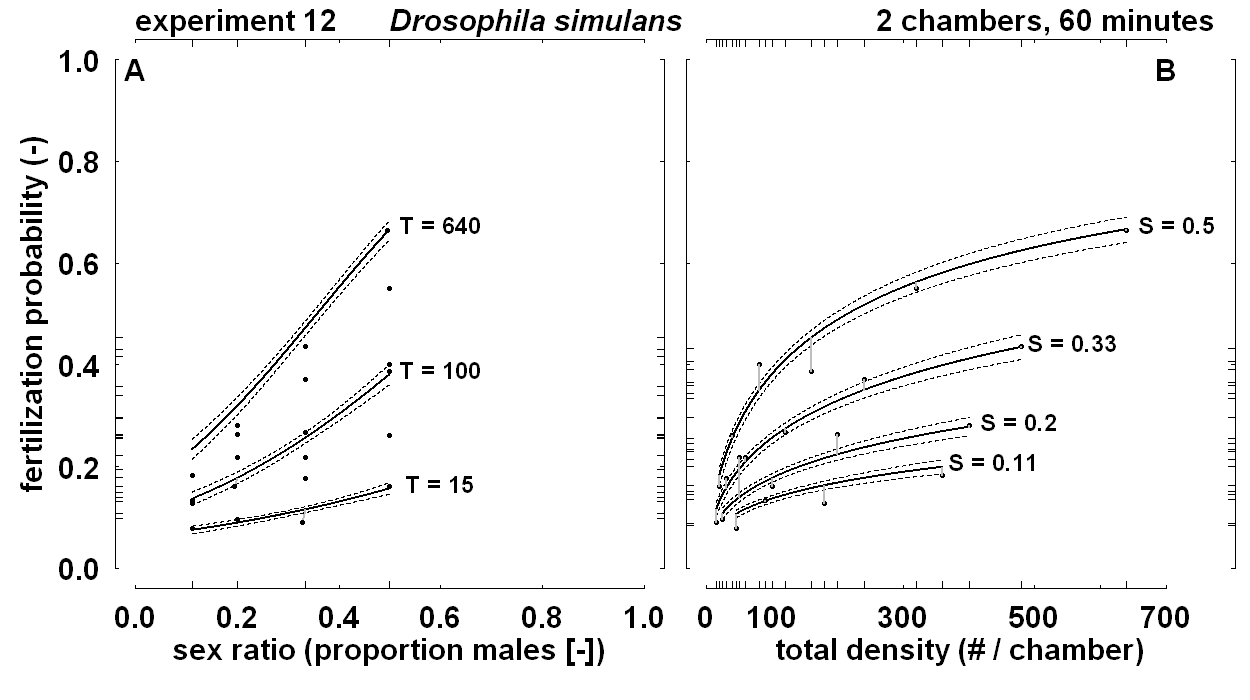
**

**Figure S4.15. Fertilization probability of female *Drosophila* *simulans* (60 minutes, two chambers).** Data is from Wallace [2] and concerns the experiment in which flies were together in two experimental chambers for 60 minutes. Composition as in Figure S4.4.

**
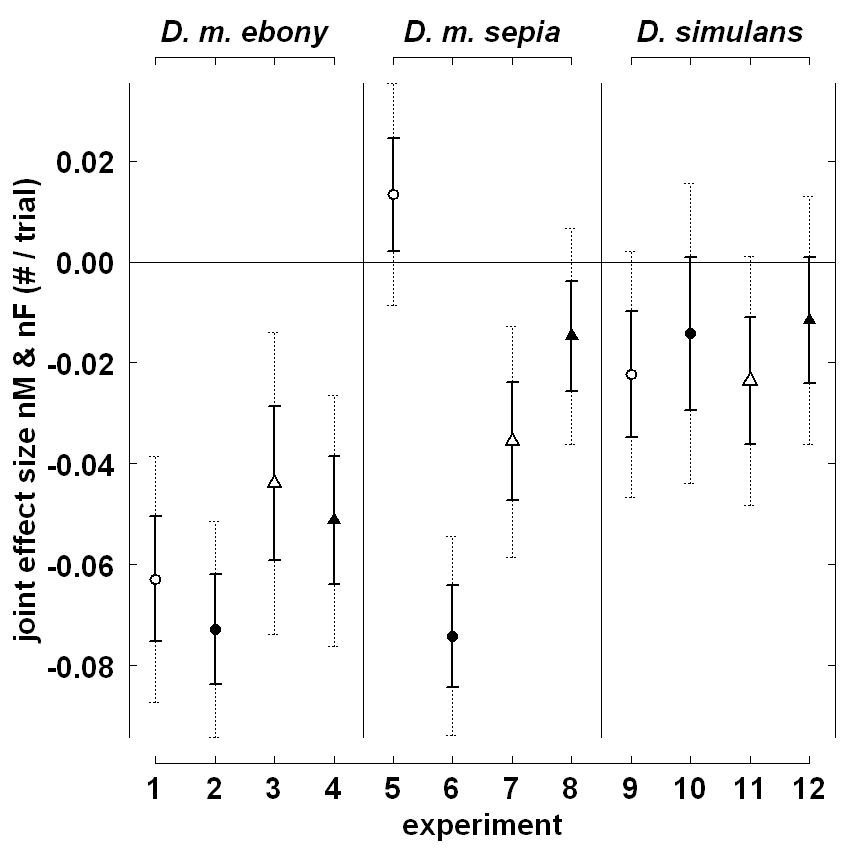
**

**Figure S4.16**. **Interaction effects** **on the ln-transformed odds (i.e. logits) of fertilization of female *Drosophila*.** Depicted are the parameter estimates of the joint effect size of the total number of male flies (nM) and female flies (nF) for each of the twelve experiments presented in Wallace [2]. Symbols indicate trial duration (30 min: open symbols, 60 min: filled symbols), and the number of mating chambers in the experiment (1: circles, 2: triangles). Solid and dotted error bars indicate the standard error and the 95% confidence intervals of these parameter estimates, respectively. Negative join effect sizes can be interpreted as indicating that the positive effect of the total number of males and the negative effect of the number of females was less pronounced when more flies of the other sex were around.
